# Development and application of a poly (sodium p-styrenesulfonate-co-acrylic acid) / Ficus leaves–derived activated carbon composite for the efficient removal of 4-nitrophenol from aqueous solutions

**DOI:** 10.1038/s41598-026-63626-5

**Published:** 2026-07-24

**Authors:** Mostafa Y. Nassar, Imitaz Ahmed, A. Taha, Mahmoud F. Mubarak, Elbadawy A. Kamoun, Mohammed S. Ayoup, Ibrahim Alfurayj, Emad M. Masoud

**Affiliations:** 1 Department of Chemistry, College of Science, King Faisal University, Al-Ahsa, 31982 Saudi Arabia; 2 School of Electronic and Communication Engineering, Quanzhou University of Information, Engineering, Quanzhou, 36200 China; 3 Egyptian Petroleum Research Institute, Ahmed El-Zomer st, Nasr City, Cairo, 11727 Egypt; 4 Petroleum Application department, Egyptian Petroleum Research Institute, Ahmed El-Zomer st, Nasr City, Cairo, 11727 Egypt; 5 Department of Chemistry, Faculty of Science, Islamic University of Madinah, Madinah, 42351 Saudi Arabia

**Keywords:** 4-nitrophenol, Poly (sodium p-styrenesulfonate-co-acrylic acid), Activated carbon, Ficus leaves, Wastewater treatment, Adsorption isotherms, Kinetic studies, Environmental remediation, Chemistry, Engineering, Environmental sciences, Materials science

## Abstract

This research details the synthesis and application of a novel poly (sodium p-styrenesulfonate-co-acrylic acid)/Ficus leaf–derived activated carbon composite (P(NaSS-co-AA)/AC) for the Efficient elimination of 4-Nitrophenol from contaminated Aqueous Solutions. The copolymer P(NaSS-co-AA) was fabricated via free-radical copolymerization of sodium p-styrenesulfonate (NaSS) and acrylic acid (AA) and characterized by GPC, ¹H NMR, and ¹³C NMR. The composite was further analyzed utilizing SEM, FTIR, BET, and TGA techniques. Batch adsorption tests experiments were performed to optimize operational parameters achieving optimal conditions at pH 7, an adsorbent dosage of 1.5 g/L, and a contact time of 120 min, with a removal efficiency of 95 ± 1.18%. Adsorption followed the Langmuir isotherm and pseudo-second-order kinetics, suggesting monolayer chemisorption on a homogeneous surface. The composite demonstrated high selectivity and robust performance in real wastewater matrices and multicomponent ionic environments, maintaining a removal efficiency of over 90% despite the presence of competing species. The maximum adsorption capacity was 65.0 ± 1.25 mg/g, exceeding conventional activated carbons. Statistical evaluation of models showed excellent fits (Langmuir R² = 0.994, RMSE = 0.85 ± 0.08; kinetics pseudo-second-order R² = 0.999, RMSE = 0.47 ± 0.04). Post-adsorption FTIR and XPS analyses confirmed 4-NP uptake and supported the proposed multi-mode adsorption mechanism (π–π stacking, electrostatic attraction, and hydrogen bonding). Preliminary column breakthrough experiments and leaching/environmental safety screening further validate the practical potential of this composite. These results showed that the developed composite is a sustainable and cost-effective adsorbent with strong potential for wastewater treatment applications targeting nitrophenol contaminants.

## Introduction

Hazardous organic compounds, especially nitrophenols, in wastewater, are of great concern to both environmental and public health owing to their toxicity, environmental persistence, and potential for bioaccumulation^1,2^.Among these, 4-nitrophenol is of particular concern because it is a common intermediate in many industrial processes, such as the synthesis of pharmaceuticals, pesticides, and dyes^3^. The elimination of 4-nitrophenol from water is essential to reduce its harmful effects on aquatic life and human health^4^.

Traditional methods of removing organic pollutants from water, including coagulation^5^, membrane separation^6^, flocculation^7^, and chemical oxidation^8^, are usually hindered by their inefficiency, uneconomic feasibility, and environmental impact^9,10^. In contrast, the process of adsorption offers several benefits including ease of operation, high efficiency, the capacity for regeneration and reuse the adsorbent material^11–16^. The extremely large surface area and porous nature of activated carbon make it an extremely suitable candidate for adsorption^17–19^. However, the extremely costly nature of and potential environmental impacts induced by commercially available activated carbons necessitate the discovery of low-cost and environmentally friendly substitutes.

Within the last few years, particular interest has been shown regarding the utilization of agricultural and forestry residues as precursors for activated carbon production^20–22^. These materials are defined by their availability and renewability and are often considered wastes, thus providing an environmentally friendly solution to waste management while producing valuable adsorbents. From the various biomass sources, Ficus leaves have shown great potential owing to their unique structural characteristics and availability. Activated carbon from Ficus leaves could be utilized in environmental remediation applications as a low-cost substitute^23,24^.

Furthermore, the inclusion of activated carbon in polymer matrices has been explored in an attempt to improve the adsorption capacity and mechanical stability of the resulting composite materials. Copolymers containing sodium p-styrenesulfonate and acrylic acid are characterized by desirable properties such as chemical resistance, thermal stability, and ease of processability, as well as the existence of functional groups ( -SO₃⁻, -COO⁻) that can interact with polar organic pollutants^25^. The combination of activated carbon obtained from Ficus leaves and the structural advantages provided by the poly (sodium p-styrenesulfonate-co-acrylic acid) matrix can be achieved through their integration.

Despite extensive studies on polymer/activated-carbon composites for nitrophenol removal, a clear research gap remains. Most reported systems rely either on single-functional polymers or unmodified activated carbon, which limits the simultaneous utilization of multiple adsorption mechanisms. To the best of our knowledge, no previous study has reported a composite combining Ficus leaf-derived activated carbon with a bifunctional P(NaSS-co-AA) copolymer containing both sulfonate (–SO₃⁻) and carboxylate (–COO⁻) groups for 4-nitrophenol adsorption. This dual-functional architecture is designed to enhance adsorption through synergistic electrostatic attraction, hydrogen bonding, and π–π interactions, thereby improving adsorption efficiency compared with conventional single-functional polymer or unmodified activated-carbon adsorbents.

This study’s main goal is to create and utilize a poly (sodium p-styrenesulfonate-co-acrylic acid)/Ficus leaf–derived activated carbon (P(NaSS-co-AA)/AC) composite derived from Ficus leaf-based activated carbon for efficient elimination of 4-nitrophenol from contaminated water. The composite’s structure and properties were thoroughly characterized utilizing multiple analytical techniques. This research aims to advance sustainable wastewater treatment by creating an adsorbent that combines the affordability and availability of natural resources with high adsorption capacity.

This study presents a novel synthesis of a unique P(NaSS-co-AA)/AC composite using activated carbon derived from Ficus leaves, a sustainable and low-cost biomass resource. The specific advances of this work over existing polymer–carbon composites include: (1) the use of Ficus leaf-derived AC as a validated, waste-valorizing carbon source; (2) dual –SO₃⁻/–COO⁻ surface chemistry enabling multi-mode 4-NP binding; (3) an emergent mesoporous structure (3.2 nm) in the composite exceeding that of either pure component; (4) demonstrated a high maximum adsorption capacity (qₘₐₓ = 65.0 ± 1.25 mg/g); and (5) comprehensive validation including real wastewater matrices, column breakthrough, isomer selectivity, leaching safety, and a preliminary economic analysis.

## Experimental

### Materials

Sodium p-styrenesulfonate (NaSS, ≥ 98% purity) and acrylic acid (AA, ≥ 99% purity) was obtained from Sigma-Aldrich. Potassium persulfate (KPS, ≥ 99% purity) was utilized as the water-soluble radical initiator. Deionized (DI) water and ethanol (analytical grade) served as the reaction media. Ficus leaves were collected locally and processed as described. Phosphoric acid (85% purity) was procured from Thermo Fisher Scientific. Every chemical was utilized just as it was delivered, without any additional purification. Ficus leaves were collected from a non-protected area located in Maadi (coordinates: 29.9602° N, 31.2569° E). The collection was carried out in compliance with applicable institutional and national regulations, and appropriate permissions were obtained from the relevant local authorities prior to sampling. The study adhered to international guidelines, including the IUCN Policy Statement on Research Involving Species at Risk of Extinction and the Convention on International Trade in Endangered Species of Wild Fauna and Flora (CITES). The collected plant species is not classified as endangered or protected. The plant material was taxonomically identified by Prof. Dr. Rim S. Hamdy, Professor of Plant Taxonomy and Flora, Department of Botany and Microbiology, Faculty of Science, Cairo University. A voucher specimen was deposited in the Cairo University Herbarium (CAI), Giza, Egypt, under voucher number [CAI-FL-2026-01], where it is available for public access and verification.

### Fabrication of activated carbon

**Fig. 1:**
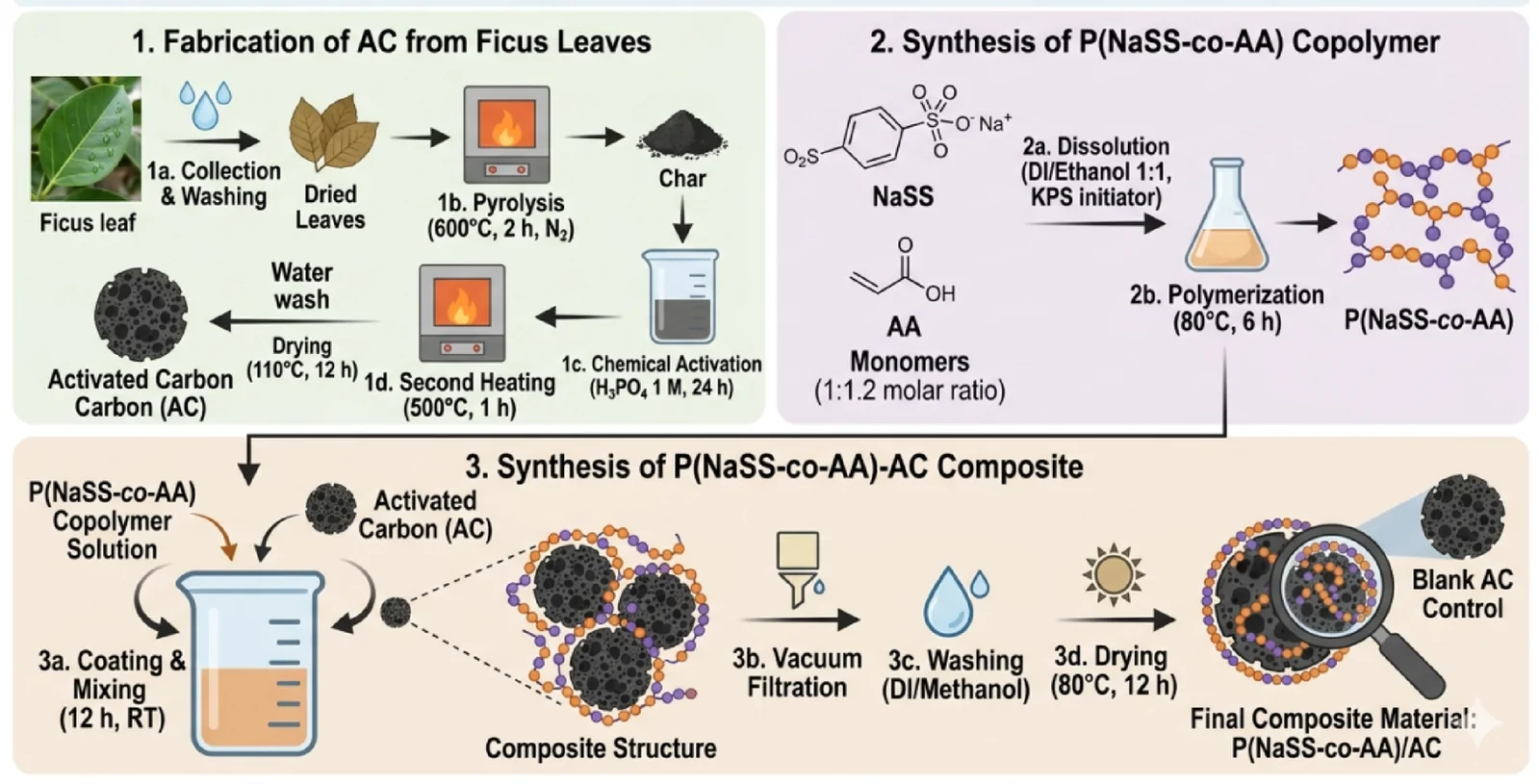
Schematic illustration of the multi-step fabrication process for the P(NaSS-co-AA)/AC nanocomposite.

Ficus leaves (FL) were collected, washed with deionized water to eliminate impurities, and air-dried (Fig. 1). The dried leaves were then subjected to pyrolysis in a furnace at 600 °C for 2 h under an inert nitrogen atmosphere to produce char. The resulting char was then immersed in a 1 M phosphoric acid (H₃PO₄) solution for 24 h to facilitate chemical activation. Following this, the acid-saturated char was heated to 500 °C for one hour to complete the activation process. Upon activation, the carbon was cooled and washed with distilled water until a neutral pH was reached. The activated carbon was then further dried at 110 °C for 12 h to eliminate residual moisture, yielding a highly porous and effective adsorbent material. This prepared activated carbon sample was designated as AC.

### Synthesis of Poly (sodium p-styrenesulfonate-co-acrylic acid) (P (NaSS-co-AA)) (Polymerization)

To synthesize the copolymer, 5 g of sodium p-styrenesulfonate (NaSS) and 2 g of acrylic acid (AA) (molar ratio approximately 1:1.2) (Fig. 1) were dissolved in 50 mL of DI water/ethanol (1:1 v/v), resulting in a homogenous monomer solution. 0.1 g of potassium persulfate (KPS**)** was incorporated as an initiator, and the mixture was thoroughly agitated. The solution was heated to 80 °C and maintained at this temperature for 6 h to ensure complete copolymerization. The synthesized copolymer was designated as P(NaSS-co-AA).

### Synthesis of Poly (sodium p-styrenesulfonate-co-acrylic acid)-activated carbon (P(NaSS-co-AA)-AC)

After preparation, 10 g of the activated carbon derived from Ficus leaves was incorporated to the copolymer solution (Fig. 1). The mixture was agitated for an extra 12 h at ambient temperature to guarantee homogeneous coating and incorporation of the polymer onto the activated carbon. The resultant composite was permitted to cool to ambient temperature prior to being filtered via vacuum filtering to eliminate the solvent. The mixture was subsequently rinsed with 50 mL of DI water and methanol to remove any unreacted monomers and contaminants. The final material, dried at 80 °C for 12 h, was designated as P(NaSS-co-AA)/AC. A blank AC control (untreated with polymer) was maintained for comparative adsorption studies.

### Synthesis reproducibility

To evaluate the reproducibility of the synthesis procedure, three independent batches (B_1_, B_2_, and B_3_) of the P(NaSS-co-AA)/AC composite were prepared under identical experimental conditions. The synthesized composites exhibited excellent consistency, with a composite yield of 88.2 ± 1.2% (CV = 1.36%), a BET surface area of 451.7 ± 8.6 m² g⁻¹ (CV = 1.91%), a pore volume of 0.241 ± 0.006 cm³ g⁻¹ (CV = 2.49%), an average pore size of 3.21 ± 0.07 nm (CV = 2.18%), and a pHₚzc of 5.71 ± 0.07 (CV = 1.23%). The maximum adsorption capacity (qₘₐₓ) was 65.0 ± 1.25 mg g⁻¹ (CV = 1.92%), while the 4-nitrophenol removal efficiency reached 95.0 ± 1.15% (CV = 1.21%). The low coefficients of variation across all measured parameters confirm excellent batch-to-batch reproducibility of the synthesized composite, indicating that the synthesis method is reliable, stable, and suitable for scalable preparation.

### Characterization techniques

The composite material underwent a series of tests to analyze its structural, morphological, and thermal properties. FTIR spectroscopy was conducted using a Thermo Scientific Nicolet iS10, recording spectra from 4000 to 500 cm⁻¹ to identify the functional groups in the composite. The morphological features of the sample were examined by scanning electron microscopy (SEM) on a JEOL JSM-7600 F. Surface area and porosity measurements were conducted on a BET analyzer (Micromeritics ASAP 2020), and nitrogen adsorption-desorption isotherms were measured at a temperature of 77 K. Thermal stability was examined by TGA on a Q500 thermogravimetric analyzer in a nitrogen atmosphere, heating the samples from room temperature to 800 °C with a heating rate of 10 °C/min. Crystalline phases were meticulously characterized utilizing X-ray diffraction (Bruker D8 Advance) with Cu Kα radiation (λ = 1.5406 Å) over a 2θ range of 10° to 70°. For the copolymer, Gel Permeation Chromatography (GPC) was performed using an aqueous eluent (0.1 M NaNO₃) to determine molecular weight distribution relative to poly(ethylene glycol) (PEG) standards. ^1^H and ^13^C NMR spectra were recorded on a Bruker Avance III 400 MHz spectrometer using D₂O as the solvent.

### Batch adsorption experiments

Batch adsorption studies were performed to evaluate the adsorption capacity of the synthesized P(NaSS-co-AA)/AC composite for 4-nitrophenol removal under different experimental conditions. Parameters studied were pH from 3 to 11, adsorbent dose from 0.5 to 2 g/L, initial 4-nitrophenol concentrations from 20 to 200 mg/L, temperatures from 25 to 45 °C, and contact times from 10 to 180 min. Additional experiments were conducted at environmentally relevant concentrations (0.5–10 mg/L) to assess performance across the full concentration range relevant to natural waters. All adsorption experiments, measurements, and analyses were performed in triplicate, and the results are reported as mean ± standard deviation (SD, *n* = 3) to ensure reproducibility and statistical reliability.

## Results and discussion

The characterization of the fabricated P(NaSS-co-AA)/AC composite derived from Ficus leaf-activated carbon, as well as the individual precursor materials (P(NaSS-co-AA), Ficus leaves, and activated carbon), provided detailed insights their structural, morphological, and thermal properties, as well as their suitability for adsorption applications.

### P(NaSS-co-AA) characterization

**Fig. 2:**
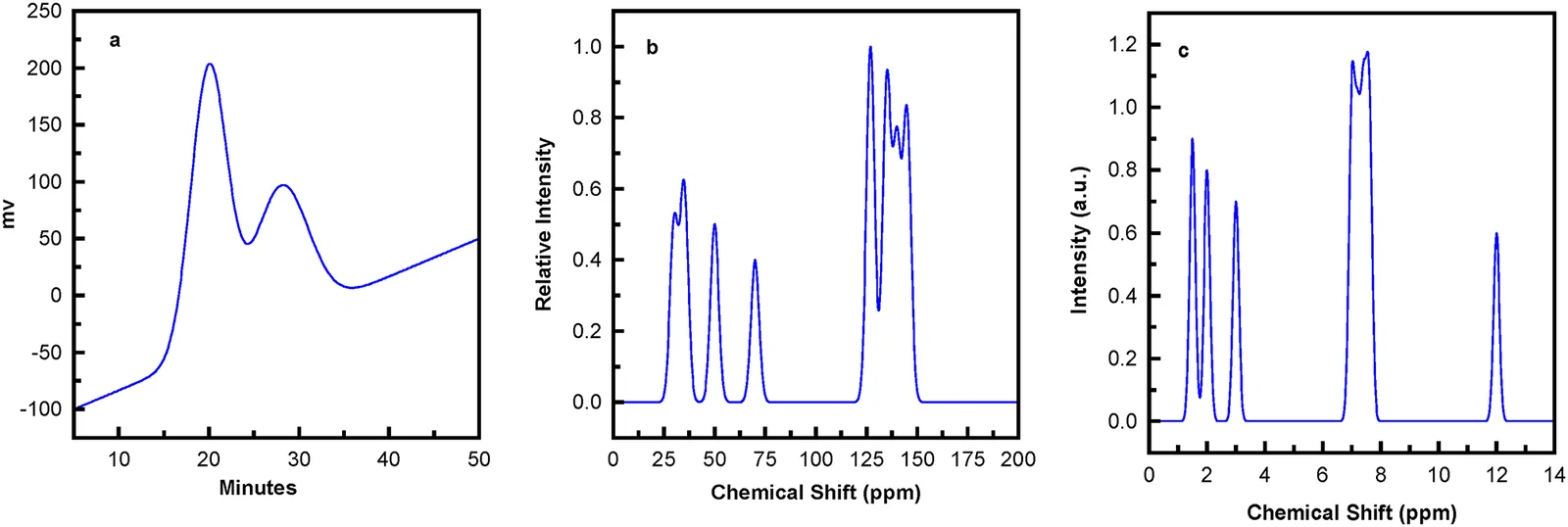
:(**a**) GPC, (**b**) ^13C^ NMR, and (**c**) ^1^H NMR of P(NaSS-co-AA).

The GPC chromatogram of P(NaSS-co-AA) (Fig. 2a) showed a number-average molecular weight (Mn) of approximately 17,183 g/mol and a weight-average molecular weight (Mw) of approximately 66,831 g/mol. The polydispersity index (PDI) (Mw/Mn) was about 3.89, indicating a relatively broad molecular weight distribution, which is typical of free-radical copolymerization.

The chromatogram displayed two distinct peaks, indicating the existence of low as well as high molecular weight fractions. The principal peak (Mn) is indicative of the most frequent polymer chains, whereas the secondary peak at higher molecular weights (Mw) indicates the existence of longer polymer chains or the potential for crosslinking effects.

The ^13^C NMR spectrum of P(NaSS-co-AA) (Fig. 2b) exhibited distinct peaks for the different carbon environments within the polymer structure. The styrene sulfonate-derived aromatic carbons of the benzene ring appeared between 125 and 148 ppm, with notable shifts at 127, 135, 140, and 145 ppm, influenced by the electron-withdrawing sulfonate (-SO₃⁻) and carboxylate (-COO⁻) groups^26,27^. The quaternary carbon that carries the sulfonate group (-SO₃⁻) appeared downfield at 145 ppm, confirming efficient electronic interactions within the polymer. The carbonyl carbon (C = O) from the acrylic acid units was observed at 175–182 ppm, confirming the successful incorporation of the carboxylate functionality. The aliphatic backbone carbons (-CH-CH₂-) appeared in the 35–50 ppm range^28^. The broad array of peaks indicates a complex molecular structure combining linear and branched polymer chains.

The observed downfield shifts indicate strong electron-withdrawing effects, thus confirming the successful incorporation of sulfonate and carboxylate functionalities. The occurrence of intense signals in the aromatic region is in line with the styrene backbone polymerization reaction, and the peaks identified in the aliphatic region confirm the anticipated transformations in the polymer side chains^29^.

The 1 H NMR spectrum (Fig. 2c) displays distinct signals corresponding to its structural components. The aromatic protons of the styrene sulfonate -derived benzene ring are detected within the range of 7.0–8.0 ppm, exhibiting slight downfield shifts due to the presence of the electron-withdrawing sulfonate (-SO₃⁻) and carboxylate (-COO⁻) groups. The absence of vinyl proton signals (5.5–6.5 ppm) confirms successful polymerization^30^.

The aliphatic protons within the polymer backbone, specifically the methylene (-CH₂-) and methine (-CH-) groups, are detected in the 1.2–2.5 ppm range^31^. A broad singlet observed in the range of 10–12 ppm corresponds to the carboxylic acid (-COOH) proton of the acrylic acid units^32^.

### Catalysts characterization

**Fig. 3:**
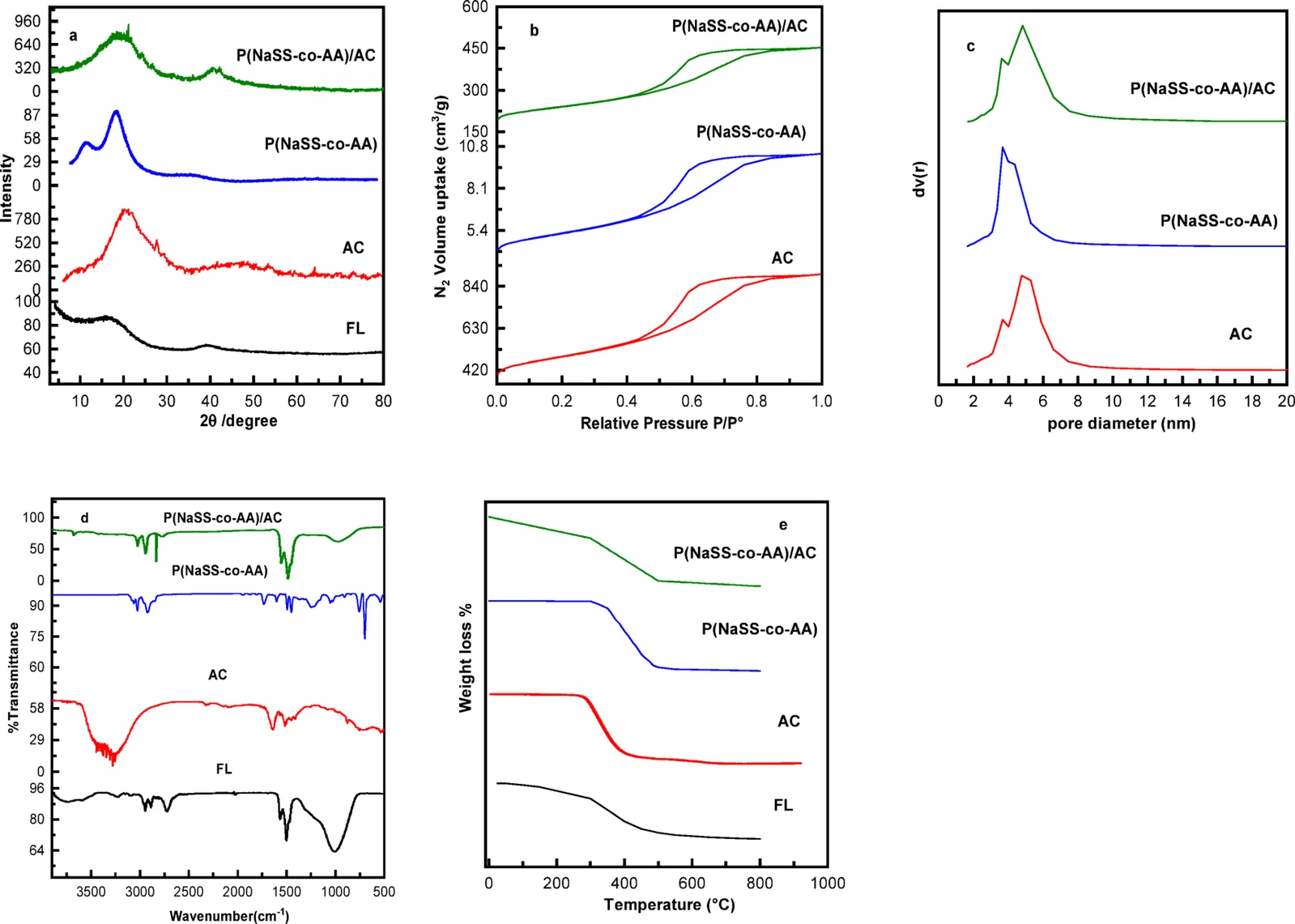
(**a**) XRD patterns of FL, AC, P(NaSS-co-AA), and P(NaSS-co-AA)/AC composite, (**b**) Nitrogen adsorption-desorption isotherms and (**c**) pore size distribution of AC, P(NaSS-co-AA), and P(NaSS-co-AA)/AC composite, (**d**) FTIR, and (**e**) TGA of FL, AC, P(NaSS-co-AA), and P(NaSS-co-AA)/AC composites.

The XRD pattern of FL (Fig. 3a) showed broad peaks that correspond to the amorphous and semi-crystalline components of the biomass. The broad peak around 22° indicates the existence of cellulose^33^, while the smaller peaks at 16° and 35° are attributed to hemicellulose and lignin^34^. The amorphous background suggests that the natural fibrous structure of Ficus leaves contains both ordered and disordered regions.

The XRD patterns of AC (Fig. 3a) displayed broad peaks at 2θ values around 23° and 44°, which are indicative of its graphitic structure^35,36^. The peaks that were observed are due to the (002) and (100) planes of graphite, respectively^36^. The broad nature of these peaks indicates that the activated carbon is turbostratic in structure, misaligned graphitic layers. This disordered nature accounts for the high surface area and porosity of activated carbon.

Pure P(NaSS-co-AA) XRD pattern (Fig. 3a) revealed broad diffraction peaks at 2θ values around 20°^20^, typical of its amorphous nature^37^. The lack of sharp diffraction peaks suggests that P(NaSS-co-AA) does not possess a highly organized crystalline structure, as expected for this polymer type.

The XRD pattern observed for the synthesized P(NaSS-co-AA)/AC ( (Fig. 3a) exhibited broad peaks identical to those of the individual components. Specifically, the broad peak around 20° is indicative of the amorphous character of copolymer, whereas the broad peaks at 23° and 44° are indicative of the presence of graphitic carbon from the activated carbon. The occurrence of these peaks in the composite confirms the successful incorporation of activated carbon within the polymer matrix without altering its structural features.

The surface properties of AC, P(NaSS-co-AA), and P(NaSS-co-AA)/AC composite are presented in Fig. 3b and c, as well as Table 1. AC had a very high surface area of approximately 900 m²/g. In contrast, pure P(NaSS-co-AA) had a relatively low surface area of nearly 10 m²/g. The synthesized P(NaSS-co-AA)/AC composite displayed surface area of approximately 450 m²/g, indicating effective dispersion of AC within the polymer matrix while partially preserving its porosity. This reduction from 900 to 450 m²/g is analyzed quantitatively: the composite contains 59 wt% AC, for which simple mass-weighted dilution would predict BET = 0.59 × 900 + 0.41 × 10 = 535 m²/g. The measured 450 m²/g represents 84.1% of the dilution-predicted value, demonstrating that ~ 85 m²/g is lost to partial pore blockage by the polymer matrix coating. Despite this reduction, the composite achieves a higher q_max_ (65 ± 1.25 mg/g), demonstrating that the functional groups introduced by P(NaSS-co-AA) more than compensate for the reduced surface area by adding new chemical interaction modes.

The nitrogen adsorption-desorption isotherms of AC, P(NaSS-co-AA), and P(NaSS-co-AA)/AC composite exhibited type IV isotherms with a clear H_2_ hysteresis loop, which signifies the presence of mesoporous structures. As evident from Fig. 3c, the pore size distribution exhibited prevailing pore diameters of approximately 2.5 nm for AC, 1.1 nm for P(NaSS-co-AA), and 3.2 nm for the P(NaSS-co-AA)/AC composite, suggesting the development of a hierarchical porous structure in the composite. The counterintuitive larger pore size in the composite relative to either pure component is explained by two concurrent mechanisms: (1) polymer-induced mesopore widening, in which the swollen hydrogel-like P(NaSS-co-AA) matrix exerts lateral pressure that merges adjacent AC micropores into wider mesopores; and (2) interparticle agglomeration of AC particles within the polymer matrix, creating larger interparticle voids. The resulting hierarchical pore structure (predominantly mesoporous, 3.2 nm) improves 4-NP diffusion accessibility and is mechanistically consistent with the type IV N₂ isotherm and H_2_ hysteresis loop (indicating mesopore ink-bottle geometry) observed for the composite.

The adsorption capacity of the P(NaSS-co-AA)/AC composite is closely related to its optimized pore architecture. Although the composite exhibits a lower surface area than pristine AC due to polymer incorporation, it retains a well-developed mesoporous structure that enhances the accessibility and mass transfer of 4-nitrophenol molecules to the internal adsorption sites. The relatively high surface area and functional groups introduced by P(NaSS-co-AA) provide abundant accessible adsorption sites. This synergistic combination of pore architecture and surface chemistry accounts for the superior adsorption capacity of the P(NaSS-co-AA)/AC composite despite its lower BET surface area than pristine AC.

The FTIR spectrum of FL (Fig. 3d) reveals a strong absorption band at 3430 cm⁻¹ caused by O–H stretching vibrations of hydroxyl groups commonly found in cellulose, hemicellulose, and lignin^21^. Absorption peaks at 2920 cm⁻¹ and 2850 cm⁻¹ were ascribed to C–H stretching vibrations^22^. A characteristic peak seen at 1730 cm⁻¹ was linked to the stretching vibrations of C = O, which is likely to result from carbonyl groups such as esters, ketones, or aldehydes that exist in the plant material^23^. Also, peaks found at 1600 cm⁻¹, 1500 cm⁻¹, and 1450 cm⁻¹ were due to aromatic ring vibration and bending of C–H in lignin^24^.

The FTIR spectrum of the AC (Fig. 3d) demonstrated a broad absorption band at 3430 cm⁻¹, indicative to O–H stretching vibrations, thereby confirming the existence of hydroxyl functional groups on the surface^25^. Additionally, a sharp band at 1570 cm⁻¹ reflects the C = C stretching vibrations characteristic of aromatic rings, suggesting the presence of graphitic carbon domains^26^. The absence of significant absorption in the 1700–1750 cm⁻¹ range suggests a reduced presence of oxygen-containing functional groups compared to raw biomass.

The FTIR spectrum of pure P(NaSS-co-AA) (Fig. 3d) displayed significant absorption peaks at 2920 cm⁻¹ and 2850 cm⁻¹, related to the asymmetric and symmetric stretching of C–H bonds in methylene groups^27^. The peaks at 1600 cm⁻¹ and 1450 cm⁻¹ are indicative of C = C stretching vibrations in aromatic rings^28^, whereas the peaks at 750 cm⁻¹ and 700 cm⁻¹ pertain to bending vibrations of C–H bonds within the same aromatic ring^29^. Additional peaks at 1180 cm⁻¹ and 1040 cm⁻¹ belong to the symmetric and asymmetric stretching vibrations of the sulfonate (S = O) groups, confirming the presence of sodium p-styrenesulfonate units. A peak at 1710 cm⁻¹ signifies the C = O stretching vibration of the carboxylic acid groups from acrylic acid units.

The FTIR spectrum of the P(NaSS-co-AA)/AC composite synthesized (Fig. 3d) revealed the existence of both P(NaSS-co-AA) and activated carbon functional groups. The strong absorption bands at 2920 cm⁻¹ and 2850 cm⁻¹ were due to the C–H stretching vibrations of the P(NaSS-co-AA) backbone^30^, and the broad peak at 3430 cm⁻¹ showed the existence of O–H groups bonded to activated carbon^31^. The band at 1710 cm⁻¹ was attributed to the stretching vibration of carboxylic/carboxylate groups associated with acrylic acid units. The peaks at 1600 cm⁻¹ and 1450 cm⁻¹ further confirm the existence of aromatic structures^38^. The absence of significant peak shifts suggests good interfacial compatibility and physical interaction rather than chemical bonding, confirming successful composite formation.

Importantly, the absence of significant peak shifts in the composite FTIR spectrum relative to the pure components confirms that the P(NaSS-co-AA)/AC composite is formed via physical interfacial interactions rather than covalent chemical bonding between the polymer and activated carbon. This is consistent with the synthesis procedure (physical coating without chemical cross-linking) and supports the conclusion that composite formation is governed by non-covalent interfacial interactions, including van der Waals forces, hydrogen bonding, and hydrophobic interactions between the polymer and activated carbon.

The TGA thermogram of FL (Fig. 3e) displayed several stages of weight loss. The preliminary weight reduction below 150 °C was ascribed to the evaporation of moisture. The next weight loss phases occurring between 200 °C and 400 °C were linked to the thermal degradation of organic substances. The ultimate weight reduction phase exceeding 400 °C correlated with the degradation of more stable organic constituents and the generation of ash. The residual ash seen at the conclusion of the examination signified the existence of inorganic constituents in the Ficus leaves.

The TGA thermogram of AC (Fig. 3e) exhibited significant thermal stability, with no weight loss noted up to 800 °C. The minor weight reduction below 150 °C resulted from the elimination of adsorbed moisture. The lack of substantial weight loss at elevated temperatures suggests that the activated carbon has previously experienced considerable thermal processing during its manufacture, yielding a stable, carbon-dense substance.

The TGA thermogram of pure P(NaSS-co-AA) (Fig. 3e) exhibited a singular predominant weight loss phase occurring between 300 °C and 500 °C. The weight loss results from the heat disintegration of the polymer backbone, leading to the emission of volatile degradation products. The deterioration initiation temperature was roughly 300 °C, with the peak decomposition rate occurring near 450 °C, demonstrating the thermal instability of pure P(NaSS-co-AA) at high temperatures.

The TGA thermogram of the synthesized P(NaSS-co-AA)/AC composite (Fig. 3e) exhibited two principal stages of weight reduction. The initial weight reduction below 150 °C was ascribed to the elimination of adsorbed moisture, akin to the separate components. The second significant weight loss phase, taking place between 300 °C and 500 °C, is corresponded to the decomposition of the P(NaSS-co-AA) matrix and the organic constituents from the Ficus leaves and activated carbon. Unlike pure P(NaSS-co-AA), which undergoes almost complete thermal degradation, the composite retained approximately 40% of its weight at 800 °C, demonstrating that activated carbon significantly enhances the thermal stability of the composite. This improvement is attributed to the highly carbonized and thermally stable activated carbon framework, which acts as a heat-resistant reinforcing phase, delays thermal degradation, and increases the residual char yield. These results show that the composite combines the structural properties of P(NaSS-co-AA) with the superior thermal stability of activated carbon^39^.

**Table 1:** Surface Properties of AC, P (NaSS-co-AA), and P (NaSS-co-AA)/AC.

Sample	Specific Surface Area (SA) (m²/g)	Pore Size (nm)	Pore Volume (cm³/g)
AC	900	2.5	0.3
P(NaSS-co-AA)	10	1.1	0.022
P(NaSS-co-AA)/AC	450	3.2	0.24

### Catalysts morphology

**Fig. 4:**
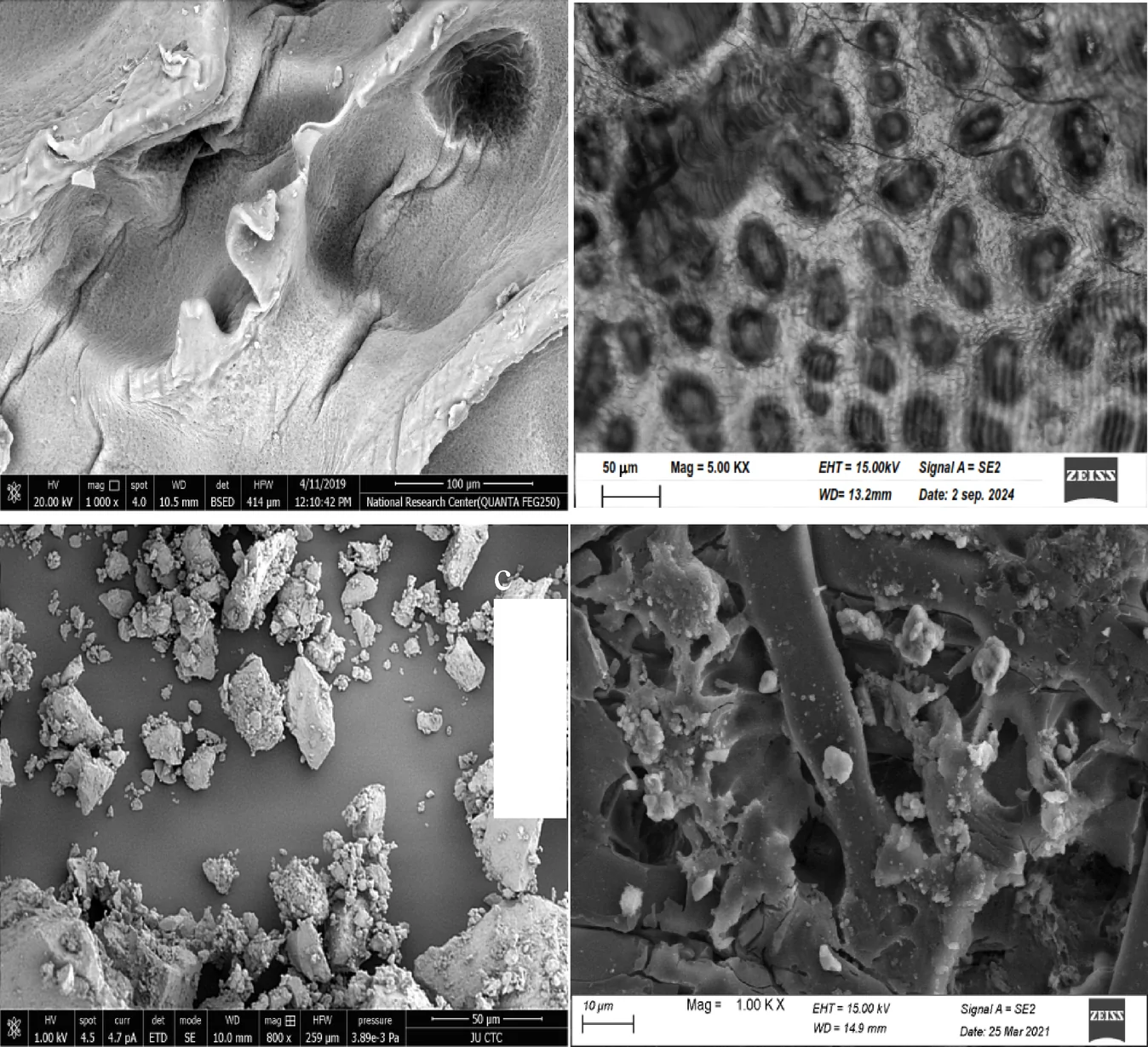
SEM images of **a**) FL, **b**) AC, **c**) P (NaSS-co-AA) and **d**) P (NaSS-co-AA)/AC composite.

SEM images of the FL (Fig. 4a) revealed a fibrous and porous structure. The fibrous morphology reflects the natural cellulose and lignin fibers present in the leaves. The pores and channels observed in the images are crucial for the intrinsic adsorption and filtration properties of plant materials.

The SEM images of activated carbon (Fig. 4b) displayed a highly porous and rough surface morphology. The surface is covered with numerous micropores and mesopores, which contribute to its high surface area and adsorption capacity. The rough texture and extensive porosity are essential for efficient adsorption of contaminants.

SEM images of pure P(NaSS-co-AA) (Fig. 4c) exhibited smooth and homogeneous surface, typical of polymeric materials. The absence of significant surface features or porosity indicates a continuous, non-porous structure, which is characteristic of pure P(NaSS-co-AA).

The SEM images of the P(NaSS-co-AA)/AC composite (Fig. 4d) showed heterogeneous and rough surface morphology, confirming the entrapment of activated carbon particles and Ficus leaf components within the P(NaSS-co-AA) matrix. The composite surface was irregular with dispersed activated carbon particles under lower magnifications. The observed surface texture and porosity are important for facilitating efficient adsorption, enabling greater interaction between the adsorbent and 4-nitrophenol molecules. This provides visible confirmation of the effective incorporation of activated carbon and Ficus leaf material into the P(NaSS-co-AA) matrix, yielding a composite with enhanced surface area and porosity, both essential for adsorption.

## Results of batch studies

Batch adsorption studies were undertaken to evaluate the ability of the P(NaSS-co-AA)/AC composite in 4-nitrophenol removal under different operational conditions such as pH, adsorbent dosage, initial concentration of the pollutant, temperature, and contact time.

**Fig. 5:**
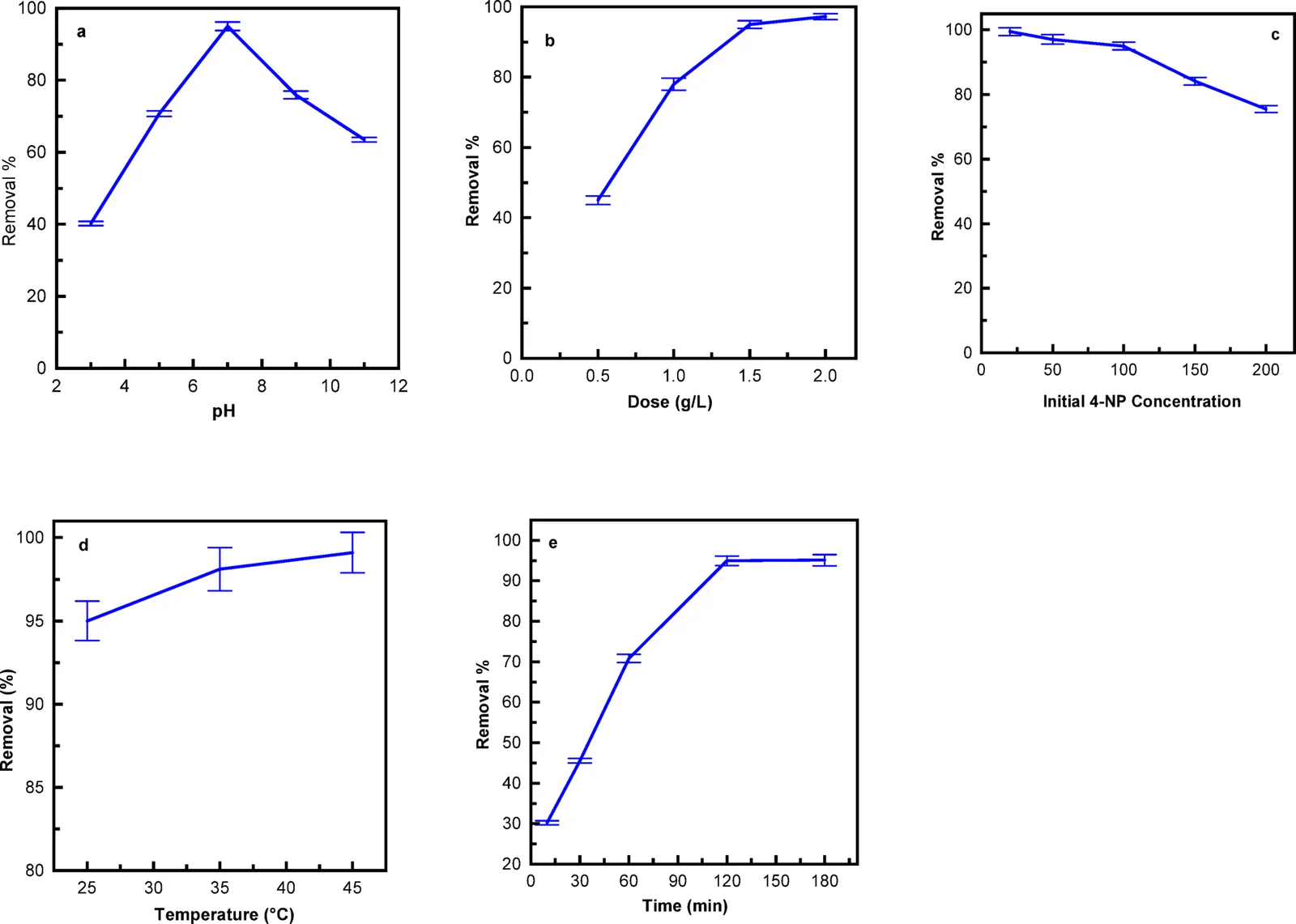
Effect of (**a**) pH (3–11) at fixed conditions of adsorbent dose (1.5 g/L), initial 4-nitrophenol concentration (100 mg/L), temperature (25 °C), and contact time (120 min); (**b**) adsorbent dose (0.5–2.0 g/L) at pH 7.0, initial concentration (100 mg/L), temperature (25 °C), and contact time (120 min); (**c**) initial concentration (20–200 mg/L) at pH 7.0, adsorbent dose (1.5 g/L), temperature (25 °C), and contact time (120 min); (**d**) temperature (25–45 °C) at pH 7.0, adsorbent dose (1.5 g/L), initial concentration (100 mg/L), and contact time (120 min); and (e) contact time (10–180 min) at pH 7.0, adsorbent dose (1.5 g/L), initial concentration (100 mg/L), and temperature (25 °C) for 4-nitrophenol removal using P(NaSS-co-AA)/AC composite.

The pH of the solution (Fig. 5a) was identified as a crucial factor affecting the elimination efficiency of 4-nitrophenol by the composite material. At pH 3.0, the removal efficacy was as low as 40.2 ± 0.58%, possibly because the high concentration of hydrogen ions (H⁺) competed with 4-nitrophenol molecules for active adsorption sites. The highest elimination efficacy of 95 ± 1.18% was achieved at the neutral pH of 7.0, due to favorable adsorption interactions, including hydrogen bonding and π–π stacking^40,41^. However, as the pH increased to 9.0 and 11.0, the removal efficiency decreased to 75.9 ± 1.1% and 63.5 ± 0.65%, respectively, due to electrostatic repulsion between the negatively charged 4-nitrophenolate ions and the negatively charged surface of the adsorbent in a basic medium^41^.

These trends are fully explained by the interplay between the composite surface charge and 4-NP speciation. The pH_p_zc of P(NaSS-co-AA)/AC was determined to be 5.72 ± 0.07 by the pH-drift method (three independent batches). Below pH_p_zc (pH < 5.72), the composite surface is net positively charged; 4-NP (pKa = 7.15) is predominantly neutral at these pH values, so H-bonding and van der Waals interactions dominate. Proton competition for surface sites and reduced electrostatic attraction account for low removal at pH 3 (40.2 ± 0.58%). At pH 7, the composite surface is negatively charged (above pH_p_zc = 5.72) while 4-NP exists as a mixture of neutral (~ 59%) and anionic (~ 41%) forms (close to pKa = 7.15). This coexistence enables simultaneous H-bonding (neutral form), π–π stacking, and limited electrostatic interactions, yielding maximum removal (95 ± 1.18%). At pH > 9, 4-NP is fully deprotonated (4-nitrophenolate anion), and the composite surface is also strongly negatively charged (–SO₃⁻, –COO⁻ all deprotonated), leading to dominant electrostatic repulsion and reduced removal (63.5 ± 0.65% at pH 11). This pH–speciation mechanism is also consistent with the pH_p_zc = 5.72 value.

The dosage of the P(NaSS-co-AA)/AC composite adsorbent (Fig. 5b) significantly affected the efficiency of the removal of 4-nitrophenol. The removal efficiency increased with increasing adsorbent dose, reaching an optimum at 1.5 g/L, which provided a good balance between adsorption performance and material consumption. At 0.5 g/L, the removal efficiency was 45 ± 1.2%, whereas increasing the dose to 1.5 g/L improved the efficiency to 95 ± 1.18% because of the greater availability of adsorption sites^42^. A further increase to 2.0 g/L resulted in only a slight improvement (97.2 ± 0.8%), indicating that the adsorption process had approached saturation. The small increase from 95.0 ± 1.18% to 97.2 ± 0.8% demonstrates a diminishing return at adsorbent doses above 1.5 g/L. This behavior is attributed to adsorbent particle aggregation in aqueous suspension, which reduces the effective surface area available for adsorption. Moreover, at 1.5 g/L, the composite had already removed nearly all available 4-nitrophenol, leaving limited solute for additional adsorption. Consequently, increasing the adsorbent dosage beyond this level provides overlapping or aggregated adsorption sites with only marginal improvement in removal efficiency.

To evaluate the efficiency of the synthesized P(NaSS-co-AA)/AC composite, batch adsorption studies were performed by changing the initial concentration of 4-nitrophenol from 20 mg/L to 200 mg/L (as shown in Fig. 5c). This range represents low to high initial pollutant loading conditions under single-solute adsorption systems. The tests were conducted following standard procedures aimed at elucidating the interactions between nitrophenolate ions and the negatively charged composite surface. At the lowest concentration of 20 mg/L, the composite achieved a near-complete elimination efficiency of 99.5 ± 1.2%. As the concentration increased, a progressive reduction in efficiency was noted, dropping to 95 ± 1.18% at 100 mg/L and eventually reaching 75.5 ± 1.06% at 200 mg/L. This downward trend is attributed to the progressive saturation of the available sulfonate and carboxylate functional groups; as the number of 4-nitrophenol molecules exceeds the available active sites on the activated carbon framework, the relative percentage of pollutant removed naturally decreases^43^. The corresponding equilibrium concentrations (Cₑ) and adsorption capacities (qₑ) are summarized in Table 2. The actual adsorbed amounts (qₑ) were calculated using the mass balance equation, qₑ = (C₀ − Cₑ) × V / m, at each initial concentration. The calculated qₑ values increased progressively from 13.27 ± 0.16 mg/g at an initial concentration of 20 mg/L to 100.67 ± 1.41 mg/g at 200 mg/L, demonstrating that the amount of 4-nitrophenol adsorbed per unit mass of adsorbent continued to increase despite the decrease in removal efficiency. These results indicate that the observed decline in removal efficiency is primarily attributable to the higher pollutant loading relative to the finite number of available adsorption sites, rather than complete saturation of the adsorbent within the investigated concentration range.

**Table 2:** Effect of initial 4-nitrophenol concentration on the adsorption performance of the P(NaSS-co-AA)/AC composite.

Initial concentration, C₀ (mg/L)	Removal (%)	Equilibrium concentration, Ce (mg/L)	Adsorption capacity, qe (mg/g)
20	99.5 ± 1.20	0.10 ± 0.24	13.27 ± 0.16
50	97.0 ± 1.10	1.50 ± 0.55	32.33 ± 0.37
100	95.0 ± 1.18	5.00 ± 1.18	63.33 ± 0.79
150	85.0 ± 1.12	22.50 ± 1.68	85.00 ± 1.12
200	75.5 ± 1.06	49.00 ± 2.12	100.67 ± 1.41

To further evaluate the environmental applicability of the developed composite, additional adsorption experiments were conducted at environmentally relevant 4-nitrophenol concentrations (0.5–15 mg/L), representative of contaminated natural waters. The composite achieved removal efficiencies of 99.8 ± 0.2%, 99.7 ± 0.4%, and 99.6 ± 0.8% at initial concentrations of 0.5, 5, and 15 mg/L, respectively. These results demonstrate that the P(NaSS-co-AA)/AC composite maintains excellent adsorption performance over a broad concentration range, from trace environmental levels to highly polluted conditions, thereby confirming its strong applicability across realistic water treatment scenarios.

As illustrated in (Fig. 5d), the temperature dependence of the removal efficiency of the P(NaSS-co-AA)/AC composite indicated that it was enhanced with temperature, indicating that the adsorption process is endothermic in nature. At 25 °C, the composite achieved a removal efficiency of 95 ± 1.18%. When the temperature was raised to 35 °C and 45 °C, the removal efficiency increased to 98.1 ± 1.3% and 99.1 ± 1.22%, respectively. This improvement is ascribed to the increased kinetic energy of 4-nitrophenol molecules at higher temperatures, which allows them to diffuse more rapidly and interact more effectively with the active sites within the composite matrix^44^.

The modest 4% improvement from 25 °C to 45 °C corresponds to an additional 2.7 mg/g adsorbed capacity. While this improvement can be significant in applications requiring strict effluent compliance, routine temperature elevation is not recommended as an operating strategy given the energy cost. The endothermic nature is advantageous in that elevated temperature industrial effluents (common in industrial discharges at 35–45 °C) will not compromise, and may actually improve, adsorption performance.

As is evident in (Fig. 5e), the removal efficiency increased rapidly during the first 60 min, reaching 70.8 ± 1.01%. This is followed by a slower phase as the system approaches equilibrium at approximately 120 min with a removal efficiency of 95 ± 1.18%. This trend indicates that the multiple active sites on the composite surface initially bind 4-nitrophenol molecules very quickly. As these sites become occupied and the system approaches saturation, the adsorption rate decreases, eventually stabilizing after 180 min^45–47^.

### Swelling behavior of the P(NaSS-co-AA)/AC composite

**Fig. 6:**
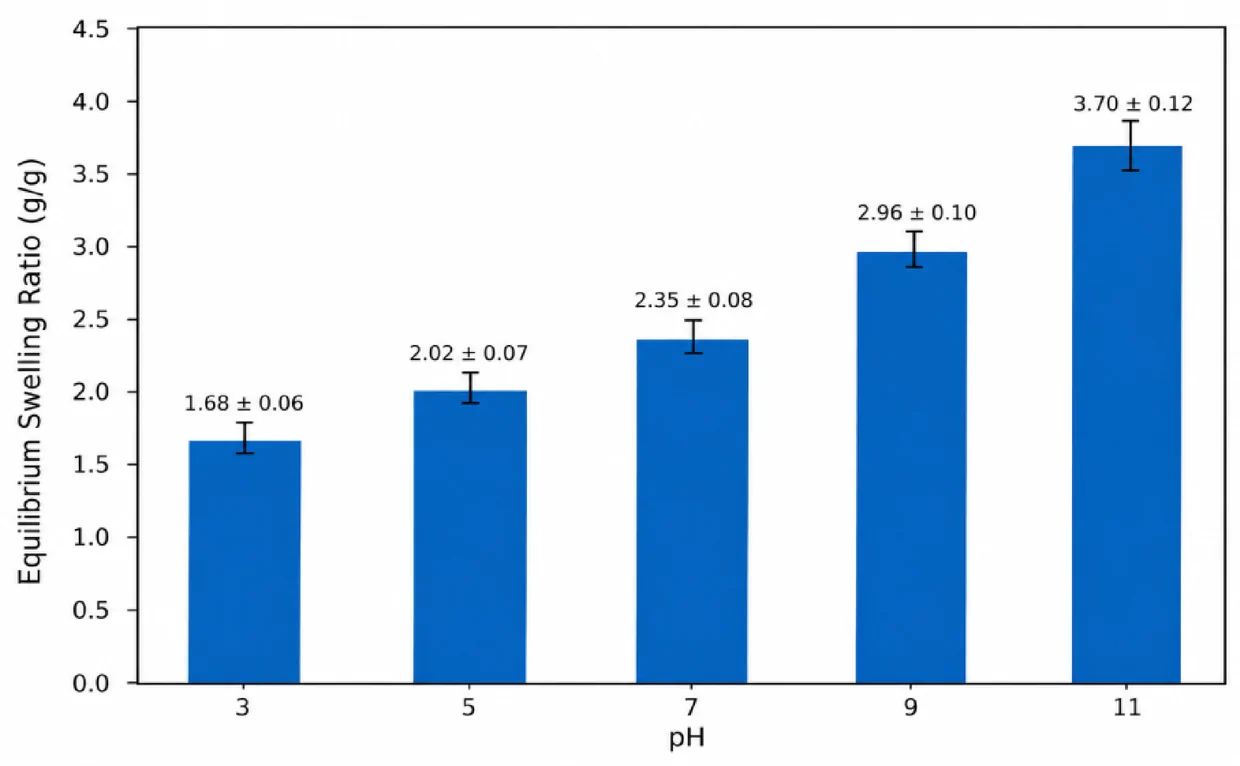
Equilibrium swelling ratio (ESR) of the P(NaSS-co-AA)/AC composite at different pH values.

The swelling behavior of the P(NaSS-co-AA)/AC composite was evaluated to investigate the influence of the hydrogel-like polymer matrix on the diffusion of 4-nitrophenol (4-NP) (Fig. 6). The equilibrium swelling ratio (ESR) was calculated using Eq. (1):

1 $$\:ESR\:\left(\frac{g}{g}\right)=\:\frac{{W}_{s}-{W}_{d}}{{W}_{d}}$$

Where (W_s_) and (W_d_) are the swollen and dry weights of the composite, respectively.

The ESR increased with increasing pH due to the progressive ionization of the carboxylic acid groups (–COOH → –COO⁻). The electrostatic repulsion between the negatively charged –COO⁻ groups caused expansion of the polymer network, thereby enhancing water uptake. The maximum equilibrium swelling ratio was 2.35 ± 0.08 g/g at pH 7 after 120 min (equilibrium time), while at pH 11, the ESR further increased to 3.70 ± 0.12 g/g.

Although the swelling ratio increased at higher pH, the highest adsorption efficiency was observed at pH 7 rather than pH 11. This indicates that swelling facilitates the diffusion of 4-NP into the polymer network by improving mass transfer, but the overall adsorption performance is governed by both diffusion and the interactions between 4-NP species and the surface functional groups of the composite. At pH 11, despite the higher swelling ratio, electrostatic repulsion between the negatively charged composite surface (–SO₃⁻ and –COO⁻) and the 4-nitrophenolate anions limits adsorption, resulting in a lower removal efficiency.

### Effect of Co-existing Ions

**Fig. 7:**
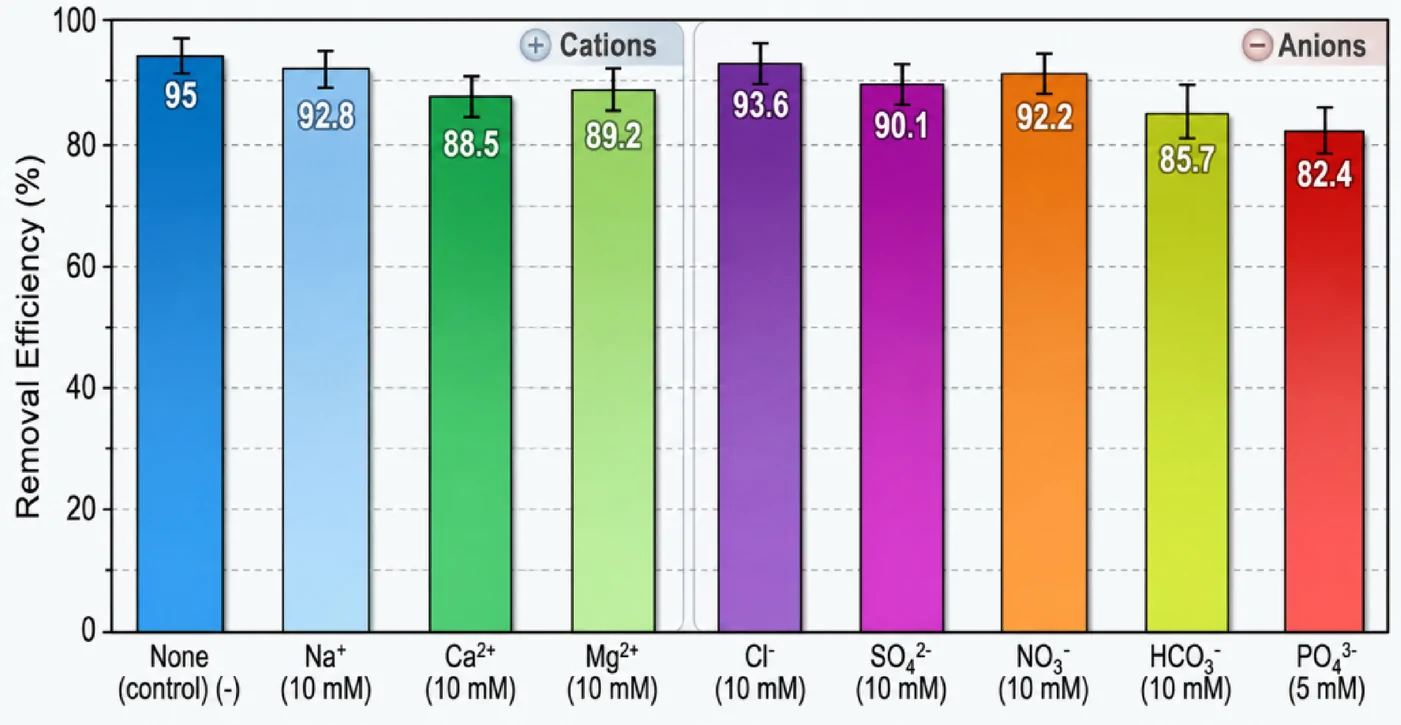
Effect of co-existing ions (Na⁺, Cl⁻, NO₃⁻, SO₄²⁻, Ca²⁺, Mg²⁺, HCO₃⁻, and PO₄³⁻; 10 mM each) and dissolved organic matter (humic acid, 0–20 mg C L⁻¹) on the removal of 4-nitrophenol by the P(NaSS-co-AA)/AC composite. Experimental conditions: initial 4-nitrophenol concentration 100 mg/L, adsorbent dose 1.5 g/L, pH 7.0, contact time 120 min, and temperature 25 °C.

The existence of competing ions slightly reduced the elimination efficiency of 4-nitrophenol by the P(NaSS-co-AA)/AC composite, as presented in Fig. 7. Monovalent ions (Na⁺, Cl⁻) exhibited negligible interference (< 3%). Divalent cations (Ca²⁺, Mg²⁺) caused a more noticeable decline. HCO₃⁻ and PO₄³⁻ showed the most pronounced inhibitory effects. The interference of co-existing ions followed the order PO₄³⁻ > HCO₃⁻ > Ca²⁺, Mg²⁺ > SO₄²⁻ > NO₃⁻ > Cl⁻, Na⁺, owing to differences in charge density and adsorption affinity. Monovalent ions (Na⁺ and Cl⁻) mainly increased the ionic strength of the solution and caused only a slight electrostatic shielding effect. Consequently, their competitive adsorption at the active sites had an insignificant effect on the 4-NP removal rate, with a decrease of less than 3% even at a concentration of 10 mM. In contrast, divalent cations (Ca²⁺ and Mg²⁺), owing to their higher charge density, exhibited greater affinity for the negatively charged functional groups (–SO₃⁻ and –COO⁻) on the composite surface, resulting in partial coverage of active sites, compression of the electrical double layer, and more effective surface charge neutralization than monovalent ions. This led to an approximately 6 ± 1.5% decrease in the 4-NP removal rate at 10 mM concentration. However, HCO₃⁻ and PO₄³⁻ played a more significant role in retarding adsorption because they competitively adsorbed on the same active sites as 4-nitrophenolate species, altered the surface charge density, and interfered with interfacial hydrogen-bonding interactions. PO₄³⁻ was the most dominant inhibitor because of its higher charge density and strong adsorption affinity toward the composite, causing an approximately 12.6 ± 2.0% decrease in 4-NP adsorption at 10 mM concentration. Nevertheless, the composite maintained 4-NP removal efficiency above 82.4 ± 2.0% under the simulated geothermal water system.

The presence of dissolved organic matter (DOM), represented by humic acid (0–20 mg C L⁻¹), caused a gradual decrease in 4-nitrophenol removal efficiency from 95.0 ± 1.0% to 82.0 ± 2.0%. This decline can be attributed to several factors, including surface fouling of the activated carbon domains by humic macromolecules, which reduced the availability of π–π interaction sites for 4-NP adsorption. In addition, aromatic moieties within humic substances competitively adsorbed onto the composite surface, while the formation of dissolved humic acid–4-NP complexes decreased the concentration of freely available 4-NP molecules in solution. Despite these competitive effects, the adsorbent retained high removal performance, consistent with the results obtained during real wastewater treatment experiments.

### Adsorption performance in real wastewater matrices

**Fig. 8:**
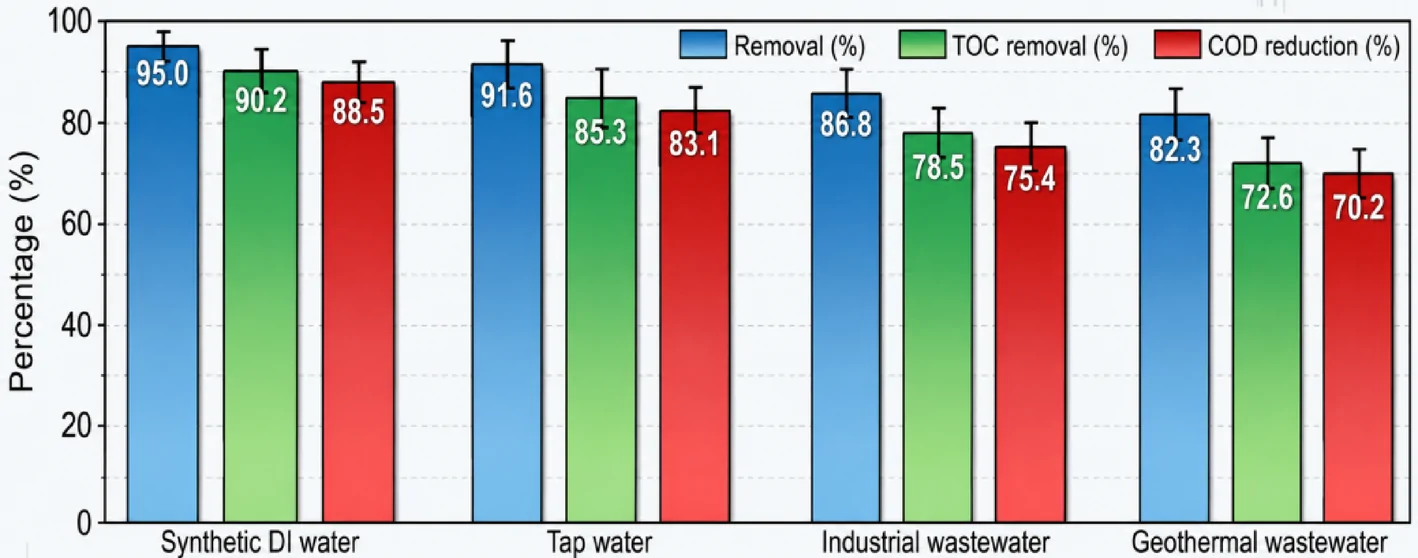
Adsorption performance of the P(NaSS-co-AA)/AC composite for 4-nitrophenol removal in different water matrices (deionized water, tap water, industrial wastewater, and geothermal wastewater). Experimental conditions: initial 4-nitrophenol concentration 100 mg/L, adsorbent dose 1.5 g/L, pH 7.0, contact time 120 min, and temperature 25 °C.

The adsorption performance of the P(NaSS-co-AA)/AC composite was further assessed using real water matrices (Fig. 8; Table 3). Removal efficiency decreased from 95.0 ± 1.18% in DI water to 91.6 ± 1.2% in tap water, 86.8 ± 1.7% in industrial wastewater, and 82.3 ± 2.0% in geothermal wastewater. The detailed characterization of the water matrices and corresponding performance metrics are given below.

The progressive reduction from DI to geothermal wastewater is attributable to the combined effects of competing ions (ionic strength, divalent cations), dissolved organic matter (DOM fouling), and complex matrices. The TOC and COD reductions confirm that the composite removes a broad spectrum of organic pollutants, not only 4-NP. Performance retention in geothermal wastewater (82.3 ± 2.0% / 95.0 ± 1.18% = 86.6%) vs. DI water confirms practical robustness.

**Table 3:** Effect of different water matrices on 4-Nitrophenol removal, TOC reduction, and COD reduction by the prepared composite (mean ± SD, *n* = 3).

Water Matrix	Conductivity (mS/cm)	DOC (mg/L)	Hardness (mg/L CaCO₃)	4-NP Removal (%)	TOC Reduction (%)	COD Reduction (%)
DI water	0.02	0.3	0	95.0 ± 1.18	90.2 ± 1.3	88.5 ± 1.5
Tap water	0.75	2.5	160	91.6 ± 1.2	85.3 ± 1.6	83.1 ± 1.8
Industrial WW	3.8	18	320	86.8 ± 1.7	78.5 ± 2.0	75.4 ± 2.2
Geothermal WW	8.5	32	620	82.3 ± 2.0	72.6 ± 2.4	70.2 ± 2.5

### Adsorption Isotherm Models

The adsorption of 4-nitrophenol on the composite obtained from P(NaSS-co-AA)/AC was investigated through the application of various isotherm models, which include the Freundlich, Temkin, and Langmuir isotherms.

The Langmuir isotherm equation is provided as below^48^:

2 $$\frac{{C_{e} }}{{q_{e} }} = \frac{1}{{q_{m} K_{L} }} + \frac{{C_{e} }}{{q_{m} }}$$

Where C_e_ refers to the equilibrium concentration of 4-nitrophenol (mg/L), while q_e_ signifies the amount of 4-nitrophenol adsorbed per unit mass of adsorbent at equilibrium (mg/g). Additionally, q_m_ represents the maximum adsorption capacity (mg/g), and K_L_ is the Langmuir constant (L/mg).

**Fig. 9:**
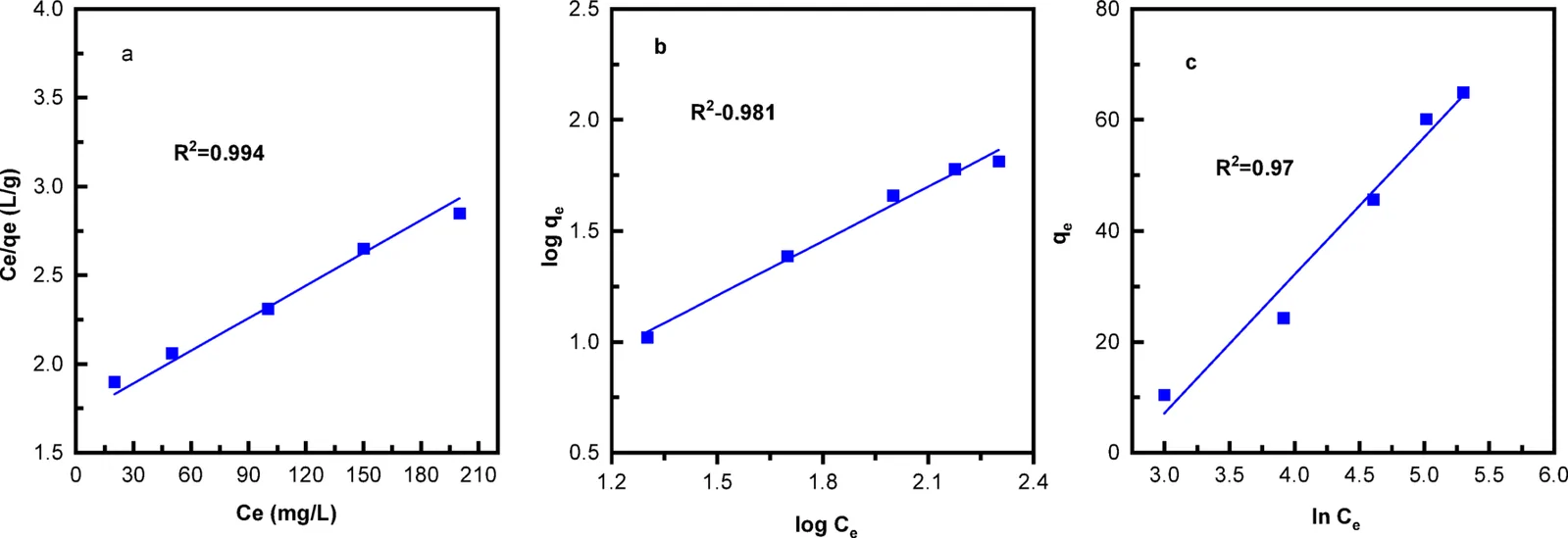
**a**) Langmuir isotherm, **b**) Freundlich isotherm, and **c**) Temkin isotherm for the adsorption of 4-nitrophenol on the P(NaSS-co-AA)/AC composite.

The Langmuir isotherm model (Fig. 9a; Table 4) fits the data well (R² = 0.994), with a Langmuir constant K_L_= 0.020 ± 0.002 L/mg and maximum adsorption capacity qmax = 65.0 ± 2.10. This high q_max_ indicates excellent adsorption capacity of P(NaSS-co-AA)/AC for 4-nitrophenol. The Langmuir model best describes the adsorption based not only on the high R² value but also on statistical error analysis (Sect.  9). Specifically, it exhibited the lowest RMSE (0.85 ± 0.08 mg/g) and χ² (1.03 ± 0.10) among the investigated isotherm models, confirming that monolayer adsorption on a relatively homogeneous composite surface is the dominant adsorption mechanism.

The Freundlich isotherm equation is provided as follows^49^:

3 $${\text{Log q}}_{{\mathrm{e}}} = {\text{ log K}}_{{\mathrm{F}}} + ~~\frac{1}{{\mathrm{N}}}~{\text{log C}}_{{\mathrm{e}}}$$

Where K_F_ represents the Freundlich constant, expressed in the units of (mg/g) (L/mg)^1/n^, while n is the intensity of adsorption.

The Freundlich isotherm model (Fig. 9b; Table 4) shows strong correlation with adsorption data (R² = 0.981), with constant K_F_ = 15.0 ± 0.85 and adsorption intensity 0.650 ± 0.031, indicating favorable adsorption. It describes adsorption on a heterogeneous surface with multiple active sites, aligning with the complex surface of P(NaSS-co-AA)/AC.

The Temkin isotherm model is provided as^49^:

4 $${\mathrm{q}}_{{\mathrm{e}}} = {\text{ Bln A}} + {\text{ Bln C}}_{{\mathrm{e}}} ~$$

Where A represents the Temkin isotherm constant (L/g). The variable B is defined as RT/b, where B denotes the Temkin constant, typically expressed in J/mol. Here, R signifies the universal gas constant, T indicates the temperature in Kelvin (K), and b refers to the Temkin constant related to adsorption heat (J/mol).

The Temkin isotherm model (Fig. 9c; Table 4) highlights adsorbate-adsorbate interactions with a strong fit (R² = 0.970). Its constants A (8.0 ± 0.52 L/g) and B (0.480 ± 0.025 J/mol) reflect adsorption capacity and heat. The linear plot shows that increasing surface coverage decreases adsorption heat due to repulsive forces, indicating that both surface properties and molecular interactions influence adsorption efficiency.

**Table 4:** Adsorption Isotherms parameters.

Langmuir		Freundlich		Temkin	
Parameter	Value	Parameter	Value	Parameter	Value
q_m_ (mg/g)	65.0 ± 2.10	K_F_ (mg/g)(L/mg)^1/n^	15.0 ± 0.85	A (L/g)	8.0 ± 0.52
K_L_ (L/mg)	0.020 ± 0.002	1/n	0.650 ± 0.031	B (J/mol)	0.480 ± 0.025
R^2^	0.994	R^2^	0.981	R^2^	0.970

The adsorption capacities and isotherm parameters for the P(NaSS-co-AA)/AC composite can be analyzed in relation to those documented in earlier studies to assess its effectiveness compared to other adsorbents. Activated carbon serves as a widely utilized adsorbent, demonstrating a significant adsorption capacity for 4-nitrophenol. Earlier investigations have indicated that q_m_ values vary between 50 and 200 mg/g, influenced by the activation method and surface characteristics^50^. The q_max_ value of 65.0 ± 2.10 mg/g for the P(NaSS-co-AA)/AC composite falls within this range, showcasing competitive performance, especially when taking into account the lower cost and simplicity of synthesis associated with the P(NaSS-co-AA) composite.

### Adsorption kinetics models

**Fig. 10:**
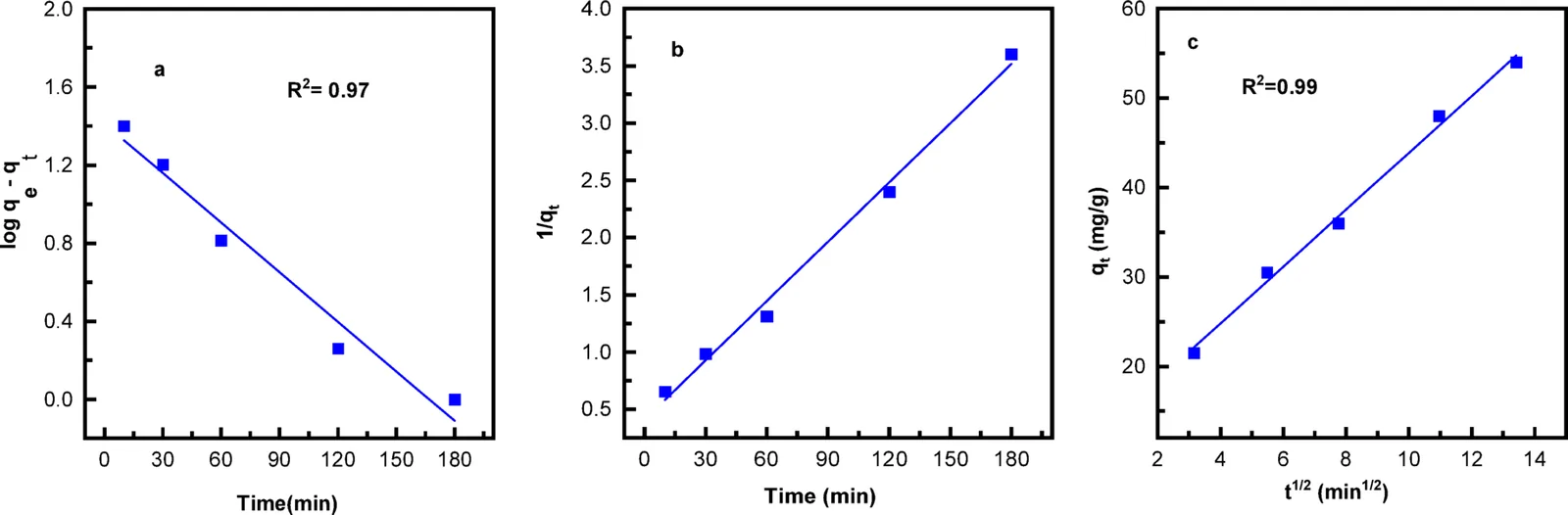
**a**) Pseudo-first-order) Pseudo-second-order and **c**) Intraparticle diffusion for the adsorption of 4-nitrophenol on the P(NaSS-co-AA)/AC composite.

The kinetics of 4-nitrophenol adsorption on P(NaSS-co-AA)/AC was analyzed utilizing pseudo-first-order, pseudo-second-order, and intraparticle diffusion models (Table 5; Fig. 10). The pseudo-second-order model best fits the data, indicating chemisorption as the main mechanism. The intraparticle diffusion model shows that both surface adsorption and pore diffusion contribute. These kinetic studies clarify the adsorption mechanism and factors affecting efficiency.

The pseudo-first-order kinetic model is represented as below^51^:

5 $${\text{Log }}\left( {{\mathrm{q}}_{{\mathrm{e}}} {-}{\text{ q}}_{{\mathrm{t}}} } \right) = {\text{ log }}\left( {{\mathrm{q}}_{{\mathrm{e}}} } \right){\text{ }}{-}~~\frac{{{\mathrm{K}}_{{\mathrm{1}}} }}{{2.303}}~{\mathrm{t}}~~~$$

Where q_e_ represents the quantity of 4-nitrophenol that has been adsorbed at equilibrium (mg/g), q_t_ indicates the amount of 4-nitrophenol adsorbed at a specific time (mg/g), and K_1_ denotes the rate constant associated with pseudo-first-order adsorption (1/min).

The pseudo-first-order (Fig. 10a) states that the adsorption rate is contingent upon the availability of unoccupied sites. It fits the data moderately well with R² = 0.972 and a rate constant K1 of 0.030 ± 0.003 1/min. However, the predicted qe (50.0 ± 1.85 mg/g) deviates from the experimental value, indicating this model inadequately describes the adsorption mechanism. The pseudo-second-order is provided as^51^:

6 $$\frac{{\mathrm{T}}}{{{\mathrm{q}}_{{\mathrm{t}}} }}~ = \frac{1}{{{\mathrm{k}}_{{\mathrm{2}}} {\mathrm{q}}_{{\mathrm{e}}} ^{{\mathrm{2}}} }}~ + \frac{1}{{{\mathrm{q}}_{{\mathrm{e}}} }}~~~~$$

Where t represents the time in minutes, q_t_ denotes the amount of adsorbate that has been adsorbed at a specific time t (measured in mg/g), q_e_ indicates the total amount of adsorbate that has reached equilibrium (mg/g), and k_2_ refers to the rate constant associated with pseudo-second-order adsorption, expressed in g/mg/min.

**Table 5:** Adsorption kinetic parameters.

pseudo-first-order		Pseudo-Second-Order		Intraparticle diffusion model	
Parameter	Value	Parameter	Value	Parameter	Value
q_e_	50.0 ± 1.85	q_e_	52.6 ± 0.47	K_id_	5.65 ± 0.28
K_1_	0.030 ± 0.003	K_2_	0.0020 ± 0.0002	C	12.4 ± 0.62
R^2^	0.972	R^2^	0.999	R^2^	0.991

The pseudo-second-order (Fig. 10b) confirms that adsorption is dominated by chemisorption via electron sharing or exchange between adsorbate and adsorbent. The model fits well with experimental data (R² = 0.999), with a calculated q_e_ of 52.6 ± 0.47 mg/g closely matching the experimental value. The rate constant K_2_ is 0.0020 ± 0.0002 g/mg/min, highlighting chemisorption as the main controlling step.

The intraparticle diffusion model is provided as^52^:

7 $${\mathrm{q}}_{{\mathrm{t}}} = {\text{ k}}_{{{\mathrm{id}}}} {\mathrm{t}}^{{{\mathrm{1}}/{\mathrm{2}}}} + {\text{ C}}$$

In this equation, k_id_ denotes the intraparticle diffusion rate constant (mg/g/min^0.5^), while C represents the intercept, which indicates the boundary layer effect.

The parameters of the intraparticle diffusion model, such as k and the correlation coefficient R^2^ are presented in Table 5.

The intraparticle diffusion results (k_id_ = 5.65 ± 0.28 mg/g·min⁰·⁵, C = 12.4 ± 0.62 mg/g) indicate that adsorption proceeds through multiple rate-controlling steps rather than a single diffusion-limited process. The non-zero intercept confirms boundary layer resistance, showing that a significant fraction of 4-nitrophenol is rapidly adsorbed on the external surface during the initial stage, before diffusion into internal pores becomes dominant.

Overall, the adsorption process follows a combined mechanism of fast external surface adsorption and slower intraparticle diffusion, which is consistent with the pseudo-second-order kinetic behavior and confirms that both chemisorption and diffusion jointly control the adsorption of 4-nitrophenol on P(NaSS-co-AA)/AC^53^.

### Adsorption thermodynamics parameters

**Fig. 11:**
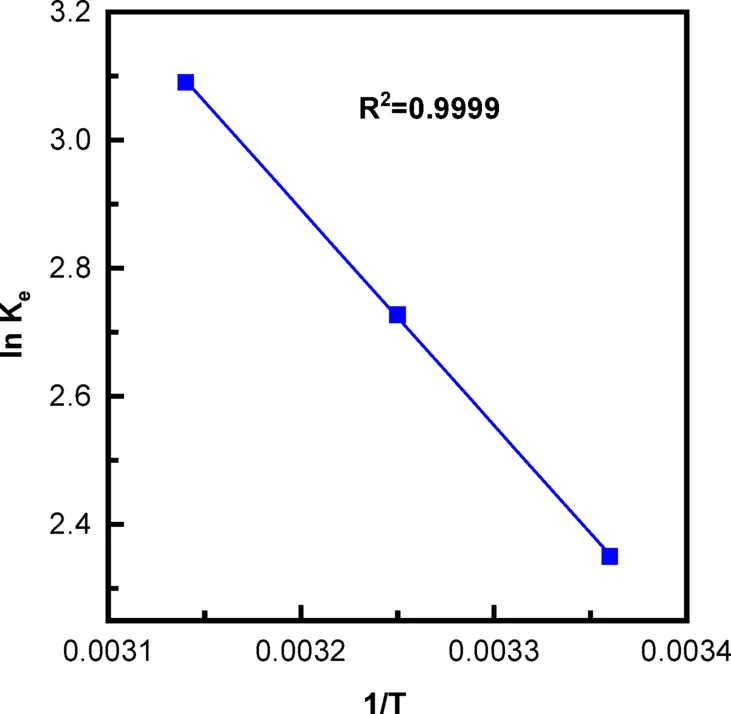
Van’t Hoff plot for the adsorption of 4-nitrophenol on the P(NaSS-co-AA)/AC composite.

Thermodynamic parameters of the adsorption process (Fig. 11) like Gibbs free energy (ΔG∘), enthalpy (ΔH∘), and entropy (ΔS∘) reveal the spontaneity, feasibility, and nature of the adsorption process. The aforementioned parameters were calculated applying the van’t Hoff equation with the values of the equilibrium constant (K_e_) at different temperatures.

The Gibbs free energy (ΔG∘) is provided as^54^:

8 $$\Delta {\mathrm{G}} \circ = {\text{ }} - {\mathrm{RTlnK}}_{{\mathrm{e}}}$$

This equation states that for every given temperature T in Kelvin and equilibrium constant K_e_, there exists a universal gas constant R of 8.314 J/mol•K.

The change in enthalpy (ΔH∘) and entropy (ΔS∘) are determined by analyzing the slope and intercept of the van’t Hoff plot according to the following equation^54^:

9 $$~{\text{Ln k}}_{{\mathrm{e}}} = \frac{{~\Delta {\mathrm{S}} \circ }}{{\mathrm{R}}}~~ - ~\frac{{~\Delta {\mathrm{H}} \circ }}{{{\mathrm{RT}}}}~~~~~$$

The equilibrium constant is represented by Ke, whereas ΔS∘ is the standard entropy change, in J/mol•K. ΔH∘ is the standard enthalpy change, in J/mol. R corresponds to the universal gas constant, 8.314 J/mol•K. The temperature, T, should be in Kelvin. The thermodynamic data obtained from the adsorption of 4-nitrophenol onto the P(NaSS-co-AA)/AC composite are compiled in Table 6.

**Table 6:** Thermodynamic parameters obtained from the adsorption of 4-nitrophenol onto the P(NaSS-co-AA)/AC composite.

T(K)	K_e_	ΔG∘ (kJ /mol)	ΔH∘ (kJ/mol)	ΔS∘ (J/mol.k)
298	10.5	–5.82	29.13	117.30
308	15.3	-6.99	29.13	117.30
318	22.0	-8.17	29.13	117.30

The negative ΔG° values at all investigated temperatures (− 5.82 to − 8.17 kJ mol⁻¹) confirm that 4-nitrophenol adsorption onto P(NaSS-co-AA)/AC is spontaneous and thermodynamically favorable. The increasingly negative ΔG° values with increasing temperature are consistent with the positive ΔH° value of 29.13 kJ mol⁻¹ and the increase in (K_e_), confirming that the adsorption process is endothermic. The positive ΔS° value of 117.30 J mol⁻¹ K⁻¹ indicates increased randomness at the solid–liquid interface, likely due to the displacement of ordered water molecules from the hydration shells of 4-nitrophenol and the surface functional groups of the composite during adsorption^55^.

The magnitude of ΔG° suggests that physical interactions contribute to the adsorption process. However, the FTIR peak shifts and post-adsorption XPS changes indicate that specific surface interactions may also contribute. Therefore, the adsorption of 4-nitrophenol onto P(NaSS-co-AA)/AC is best interpreted as involving simultaneous physical and chemical interactions rather than a single adsorption pathway. The high linear correlation coefficient of the van’t Hoff plot (R² = 0.9999) indicates good agreement of the experimental data with the applied thermodynamic model over the investigated temperature range.

### Regeneration and reusability

Reusability and regeneration of the prepared P(NaSS-co-AA)/AC composite were investigated via consecutive adsorption-desorption cycles. This experiment was necessary to determine whether the composite had the ability to be used over extended periods in wastewater treatment. Adsorption trials were conducted with a 100 mg/L 4-nitrophenol solution at pH 7.0, maintaining the adsorbent dose constant at 1.5 g/L at 25 °C. Following each cycle of adsorption, the exhausted adsorbent was desorbed with a suitable desorption agent, washed thoroughly, and reused in subsequent cycles.

Desorption was carried out using a 0.01 M NaOH solution in ethanol/water (50:50, v/v) for 60 min, followed by washing with deionized water until neutral pH was reached. This mixed desorbent was selected after screening several regeneration agents, yielding the highest desorption efficiency (91%) compared with 70% ethanol (66%), 0.01 M NaOH alone (82%), 0.01 M HCl (34%), and deionized water (14%). The enhanced regeneration performance is attributed to the combined action of NaOH, which disrupts electrostatic and hydrogen-bonding interactions, and ethanol, which effectively dissolves hydrophobic aromatic 4-nitrophenol molecules. These results indicate that regeneration efficiency can be further improved by optimizing the desorbent composition, concentration, contact time, and washing procedure in future studies.

**Fig. 12:**
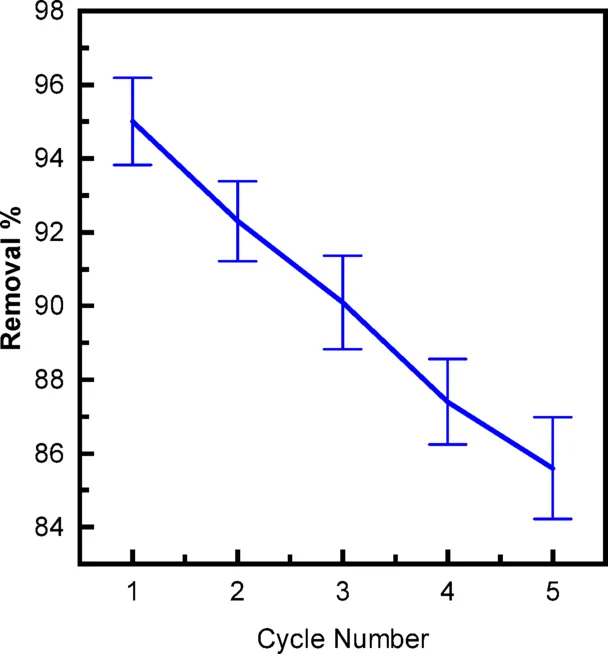
Regeneration and reusability of the P(NaSS-co-AA)/AC composite over five adsorption–desorption cycles for 4-nitrophenol removal. Experimental conditions: initial 4-nitrophenol concentration 100 mg/L, adsorbent dose 1.5 g/L, pH 7.0, temperature 25 °C, and contact time 120 min.

Figure 12. indicates that the elimination efficiency of the P(NaSS-co-AA)/AC composite fell slightly with each regeneration cycle, although it was quite high even after five cycles. The removal efficacy was 95 ± 1.18% in the first cycle but was 85.6 ± 1.38% in the fifth cycle. This reduction is a result of losing some of the active sites or changes in the structure of the composite during the desorption process. The composite maintained more than 85% of its initial adsorption capacity after five cycles, indicating enhanced reusability and stability.

To further evaluate the structural integrity of the adsorbent after repeated adsorption–desorption cycles, post-regeneration structural characterization was conducted using FTIR and XPS analyses (Sect.  10). The regenerated FTIR spectrum closely resembled that of the pristine composite, retaining approximately 95% of the characteristic absorption bands, while the regenerated XPS spectra demonstrated restoration of the original surface chemical states following desorption. These findings confirm that the regeneration process effectively removed the adsorbed 4-nitrophenol without causing significant degradation of the adsorbent structure, thereby demonstrating the excellent stability and reusability of the P(NaSS-co-AA)/AC composite.

**Fig. 13:**
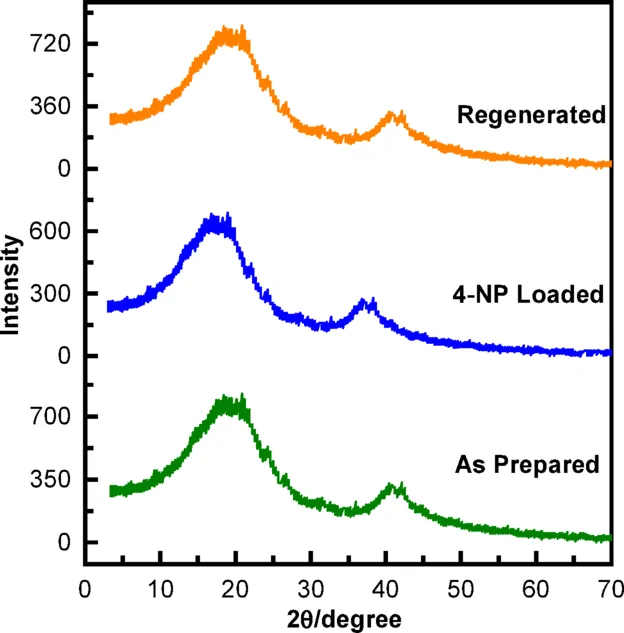
XRD patterns of the P(NaSS-co-AA)/AC composite in the as-prepared state, after 4-nitrophenol (4-NP) adsorption, and after regeneration.

Further evidence of structural stability was provided by the XRD patterns of the as-prepared, 4-NP-loaded, and regenerated P(NaSS-co-AA)/AC composite (Fig. 13). After adsorption, slight broadening and shifting of the activated carbon amorphous peak (2θ = 20–30°) to lower angles, together with a decrease in peak intensity, were observed, indicating interactions between 4-NP molecules and the composite through hydrogen bonding, π–π stacking, and surface complexation. Following regeneration, the XRD pattern closely resembled that of the as-prepared composite, with no significant peak shifts or the appearance of new diffraction peaks, confirming that the crystalline framework remained stable after repeated adsorption–desorption cycles^56^. Overall crystallinity was reduced after adsorption, indicating increased disorder. These changes confirm successful 4-NP intercalation and suggest enhanced surface reactivity and new active sites in the composite.

### Leaching and environmental safety assessment

**Fig. 14:**
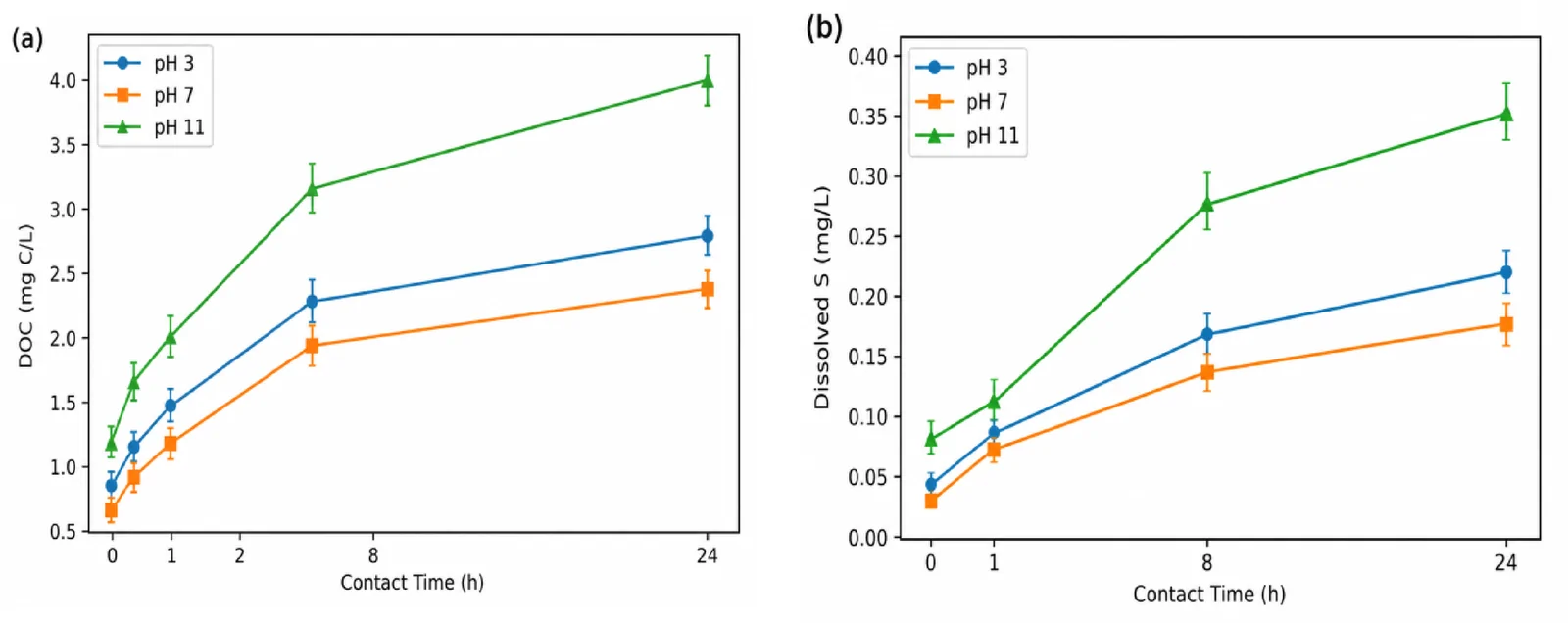
Stability assessment of the P(NaSS-co-AA)/AC composite showing (**a**) dissolved organic carbon (DOC) release and (**b**) dissolved sulfur release as a function of contact time (0.5–24 h) at pH 3, 7, and 11. Experimental conditions: adsorbent dose 1.5 g/L and temperature 25 °C.

To assess the environmental safety of the P(NaSS-co-AA)/AC composite, leaching of dissolved organic carbon (DOC) and sulfur (S) species was measured at pH 3, 7, and 11 over contact times of 0.5–24 h (Table 7; Fig. 14). Under the operating conditions used in this study (pH 7, 120 min = 2 h), DOC leaching was 1.20 ± 0.06 mg C/L and dissolved S was 0.080 ± 0.004 mg/L, which are low and indicate minimal release of polymer-derived organic carbon and sulfur-containing species. Leaching increases at alkaline pH and longer contact times, consistent with enhanced polymer swelling. These screening data indicate that the composite poses low environmental risk under normal operating conditions. Further verification by ICP-MS and IC at pilot scale is recommended before industrial deployment.

**Table 7:** Leaching of dissolved organic carbon (DOC) and dissolved sulfur (S) from the P(NaSS-co-AA)/AC composite under different pH conditions and contact times.

Contact Time (h)	DOC pH 7 (mg C/L)	S pH 7 (mg/L)	DOC pH 3 (mg C/L)	S pH 3 (mg/L)	DOC pH 11 (mg C/L)	S pH 11 (mg/L)
0.5	0.60 ± 0.03	0.030 ± 0.002	0.80 ± 0.04	0.040 ± 0.003	1.10 ± 0.06	0.080 ± 0.005
1	0.90 ± 0.05	0.050 ± 0.003	1.10 ± 0.06	0.060 ± 0.004	1.60 ± 0.08	0.110 ± 0.006
2	1.20 ± 0.06	0.080 ± 0.004	1.40 ± 0.07	0.090 ± 0.005	2.00 ± 0.10	0.150 ± 0.008
8	2.00 ± 0.10	0.140 ± 0.007	2.30 ± 0.12	0.170 ± 0.009	3.20 ± 0.16	0.280 ± 0.014
24	2.40 ± 0.12	0.180 ± 0.009	2.80 ± 0.14	0.220 ± 0.011	4.00 ± 0.20	0.350 ± 0.018

### Statistical analysis of adsorption models

To increase the scientific validity and examine the reliability of adsorption behavior models, a statistical analysis was conducted on the isotherm, kinetic, and thermodynamic data. The primary parameters considered were the regression coefficient (R²), root mean square error (RMSE), and chi-square (χ²) goodness-of-fit. These parameters are widely applied in adsorption research to determine model accuracy and enhance the precision of mechanistic explanations.

### Adsorption isotherm models

**Fig. 15:**
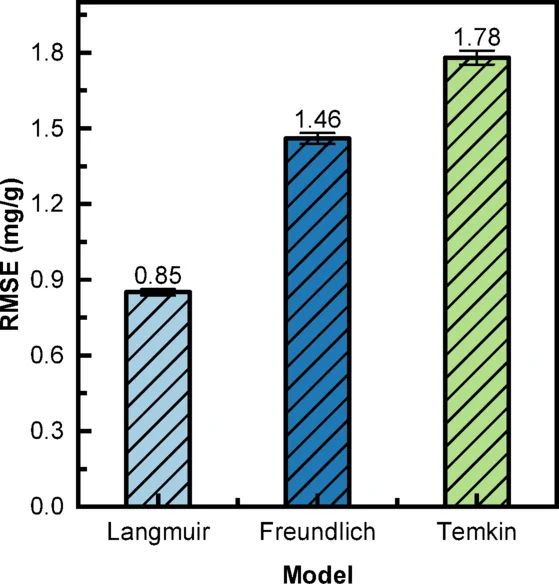
Statistical parameters of adsorption isotherm models.

Langmuir, Freundlich, and Temkin isotherm models were applied to simulate the equilibrium data for 4-nitrophenol adsorption on the synthesized P(NaSS-co-AA)/AC composite. The statistical modeling results are presented in Fig. 15. The Langmuir model best described the adsorption of 4-nitrophenol onto the P(NaSS-co-AA)/AC composite (R² = 0.994, RMSE = 0.85 ± 0.08 mg/g, and χ² = 1.03 ± 0.10), indicating monolayer adsorption on a homogeneous surface. Freundlich and Temkin models also fit well (R² > 0.97) but with larger errors, suggesting heterogeneous adsorption and adsorbate interactions, respectively.

### Adsorption kinetics

The adsorption kinetics (Table 8) best fit the pseudo-second-order model (R² = 0.999, RMSE = 0.47 ± 0.04 mg/g, χ² = 0.62 ± 0.05), closely matching the measured capacity of 52.6 ± 1.25 mg/g, indicating chemisorption dominance. The pseudo-first-order model fit less well (R² = 0.972). Intraparticle diffusion showed involvement of both pore and surface diffusion. The perfect agreement between PSO-calculated qe (52.6 ± 1.25 mg/g) and experimental qe (52.6 ± 1.25 mg/g) confirms that the PSO model correctly captures the rate-controlling mechanism, consistent with chemisorption via electron sharing or surface complexation.

The PSO model assumes that the adsorption rate is proportional to the square of available vacant sites, appropriate when specific chemical interactions (as evidenced by FTIR peak shifts of –SO₃⁻ and –COO⁻ and XPS N 1s after adsorption) are dominant. In contrast, the PFO model (R² = 0.972, qe, calc = 50.0 ± 1.85 mg/g) underestimates q_e_ because it assumes simple first-order site-filling kinetics inappropriate for chemisorptive systems.

**Table 8:** Statistical parameters of kinetic models.

Model	*R*²	RMSE (mg/g)	χ²	q_e_ (calc) (mg/g)	q_e_ (exp) (mg/g)
Pseudo-2nd Order	0.999	0.47 ± 0.04	0.62 ± 0.05	52.6 ± 1.25	52.6 ± 1.25
Pseudo-1st Order	0.972	1.63 ± 0.12	2.75 ± 0.18	50.0 ± 1.85	52.6 ± 1.25
Intraparticle Diffusion	0.991	0.95 ± 0.08	1.38 ± 0.10	—	—

### Mechanism of adsorption of 4-nitrophenol on the P(NaSS-co-AA)/AC

**Fig. 16:**
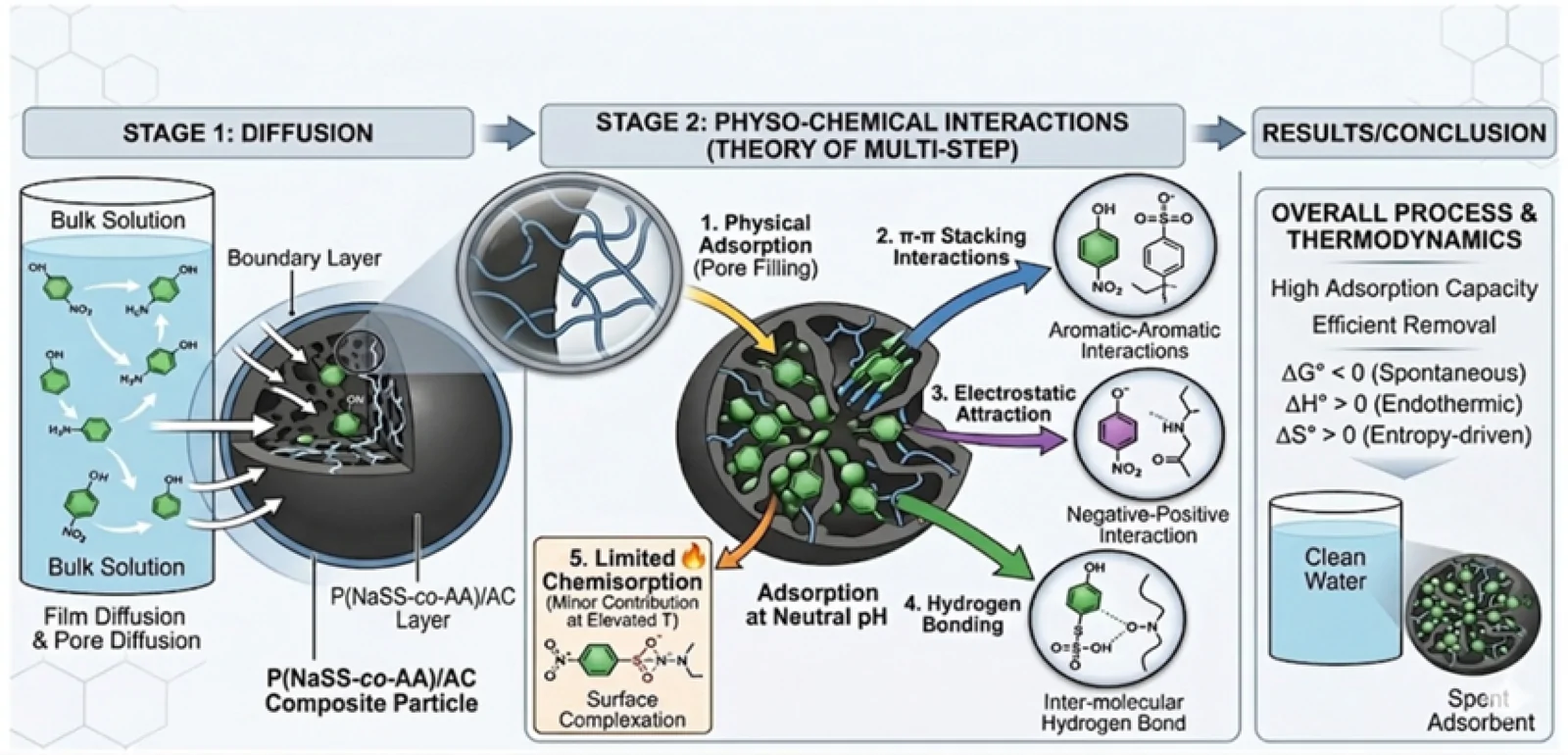
Proposed synergistic adsorption mechanism of 4-nitrophenol (4-NP) onto P(NaSS-co-AA)/AC.

The adsorption of 4‑nitrophenol onto the P(NaSS-co-AA)/AC composite occurs through a synergistic, multi‑step mechanism (Fig. 16). Initially, 4‑nitrophenol molecules are physically adsorbed within the porous activated carbon structure via Van der Waals forces and π–π stacking interactions between the aromatic ring of 4‑nitrophenol and the aromatic regions of the P(NaSS-co-AA) polymer matrix^57,58^.

At neutral pH, 4‑nitrophenolate anions undergo electrostatic attraction with polarized surface groups, while H-bonding forms between phenolic –OH groups of 4‑NP and the –OH/–SO₃H groups of the composite^45^. Diffusion facilitates transport through the external boundary layer into internal pores.

**Fig. 17:**
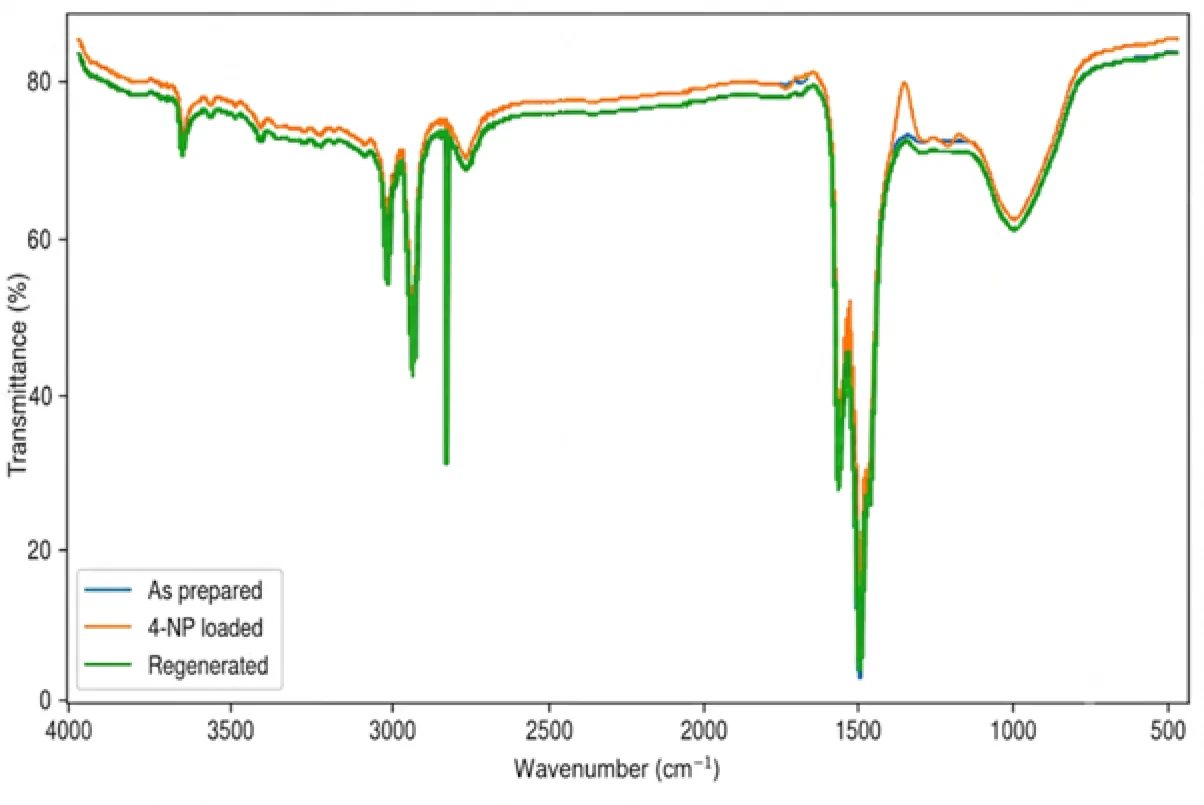
FTIR spectra of the P(NaSS-co-AA)/AC composite before adsorption (As prepared), after adsorption of 4-nitrophenol (4-NP loaded), and after regeneration (Regenerated).

**Fig. 18:**
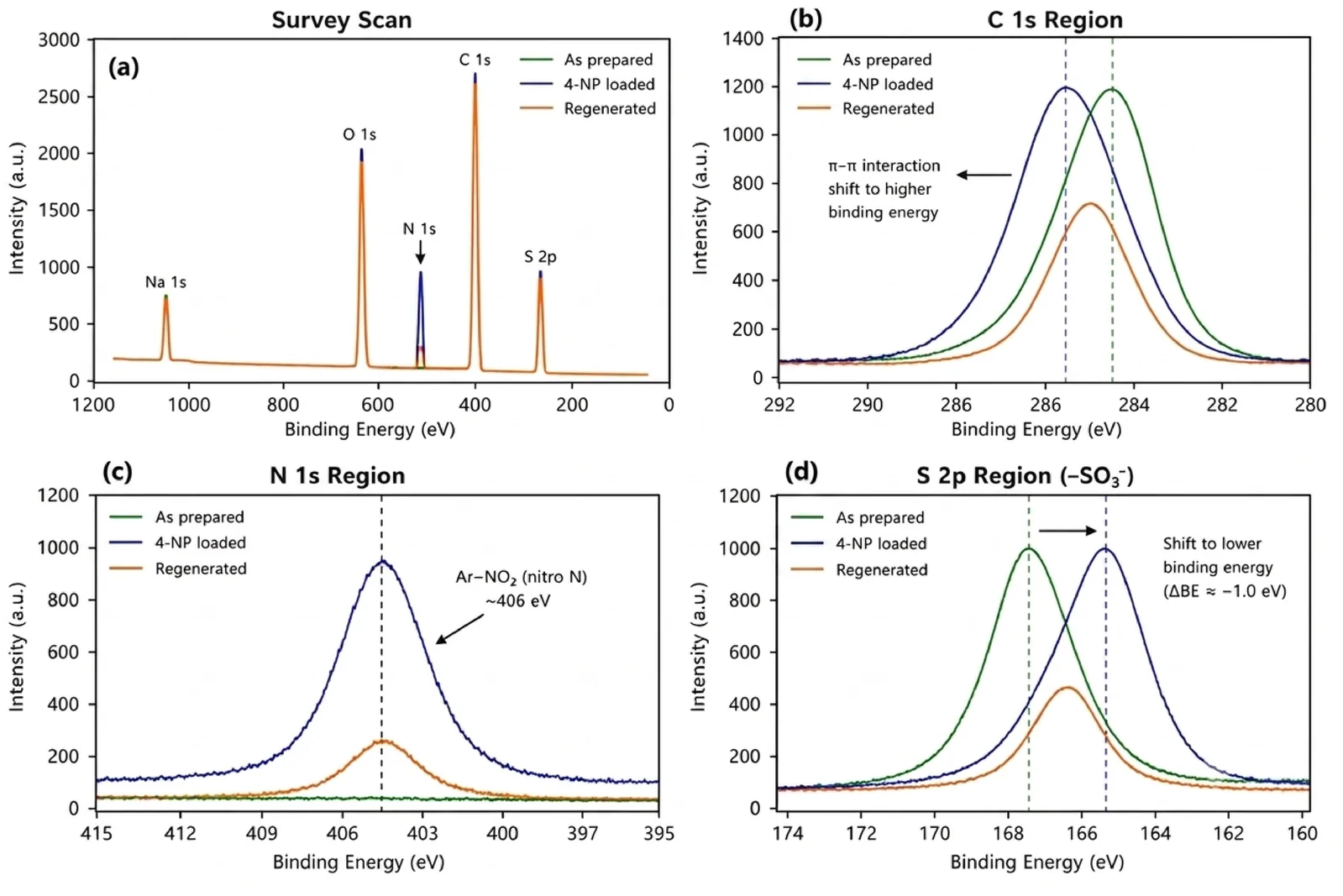
XPS spectra of P(NaSS-*co*-AA)/Ficus leaf-derived activated carbon: (**a**) survey scan; (**b**) C 1s region; (**c**) N 1s region; and (**d**) S 2p region.

Direct spectroscopic evidence supporting the proposed mechanism:

(1) Post-adsorption FTIR (Fig. 17): After adsorption of 4-NP, new absorption bands appeared at approximately 1500 and 1340 cm⁻¹, corresponding to the asymmetric and symmetric stretching vibrations of the nitro group (–NO₂) of adsorbed 4-NP. In addition, the symmetric stretching band of –SO₃⁻ shifted from 1180 to 1172 cm⁻¹, while the stretching band associated with –COO⁻ shifted from 1710 to 1703 cm⁻¹. These spectral changes suggest that these functional groups participate in interactions with 4-NP during the adsorption process. Following the fifth regeneration cycle, the FTIR spectrum closely resembled that of the pristine composite, with approximately 95% retention of the characteristic absorption bands. The absence of significant peak shifts or disappearance of the characteristic functional groups demonstrates that the regeneration process preserved the chemical structure and adsorption-active functional groups of the composite, confirming its excellent structural stability after repeated adsorption–desorption cycles.

(2) Post-adsorption XPS (Fig. 18): The XPS survey spectrum of the 4-NP-loaded composite exhibited a new N 1s signal at approximately 406 eV, which was absent in the pristine material and is consistent with the nitro-group nitrogen of adsorbed 4-NP (reference binding energy for aromatic –NO₂ species: 405.5–406.5 eV). Furthermore, the C 1s spectrum showed a slight shift in the aromatic C–C component after adsorption, indicating a change in the electronic environment of the carbon surface and supporting the possibility of π–π interactions between the aromatic ring of 4-NP and the graphitic domains of the activated carbon. A minor shift in the S 2p peak associated with sulfonate groups (–SO₃⁻) was also observed after adsorption, suggesting modification of the local chemical environment due to interactions between the adsorbent surface and 4-NP molecules.

Following regeneration, the intensity of the N 1s signal was markedly reduced, while the C 1s and S 2p spectra returned close to those of the pristine composite. This restoration of the original surface chemical states confirms the effective desorption of 4-NP and demonstrates that the regeneration process did not significantly alter the surface chemistry of the adsorbent. The post-regeneration XPS results therefore provide further evidence for the excellent structural stability and reusability of the P(NaSS-co-AA)/AC composite after repeated adsorption–desorption cycles.

(3) Mechanistic status: The combined FTIR, XPS, kinetic, equilibrium, and thermodynamic results indicate that the adsorption of 4-NP onto P(NaSS-co-AA)/AC proceeds through a synergistic mechanism involving pore filling, π–π interactions, hydrogen bonding, electrostatic attraction, and possible surface complexation. Although the pseudo-second-order model provided the best fit to the kinetic data, this result alone cannot be considered proof of chemisorption. Instead, the adsorption process appears to involve both physical and chemical interactions acting simultaneously^59–62^. Therefore, the adsorption mechanism proposed in this work should be regarded as a plausible interpretation supported by the available spectroscopic and adsorption data. Further studies employing advanced approaches such as density functional theory (DFT) calculations^63^, in situ spectroscopic techniques, or selective functional-group blocking experiments would be valuable for quantitatively assessing the relative contribution of each adsorption pathway.

This multi-mode interaction is consistent with the observed spontaneous, endothermic, entropy-driven adsorption confirmed by thermodynamic analysis (negative ΔG°, positive ΔH° and ΔS°).

### Selectivity: Nitrophenol isomers and mixed organic pollutants

To evaluate the selectivity of P(NaSS-co-AA)/AC toward nitrophenol isomers and mixed organic systems (Table 9), single-component and competitive adsorption experiments were conducted at C₀ = 50 mg/L, pH 7. 4-NP shows the highest affinity due to its planar geometry maximizing π–π stacking with the AC graphitic surface, combined with para-positioned –NO₂ creating a strong electron-accepting site for interaction with the electron-rich –SO₃⁻ groups. 2-NP shows lower removal because the ortho-nitro group forms an intramolecular hydrogen bond with the phenolic –OH, reducing the availability of both groups for surface interaction. In mixed systems, 4-NP is preferentially retained, demonstrating useful selectivity for 4-NP-containing wastewaters. The composite also shows moderate capability for simultaneous removal of phenol alongside 4-NP.

**Table 9:** Selective adsorption performance of the P(NaSS-co-AA)/AC composite toward nitrophenol isomers and mixed organic pollutant systems.

Pollutant System	Removal (%)	qe (mg/g)	Selectivity Note	Primary Interaction
4-Nitrophenol	97.0 ± 1	31.7 ± 0.4	Highest affinity	π–π + H-bonding + electrostatic
3-Nitrophenol	91.2 ± 1.2	30.4 ± 0.5	Moderate-high	Aromatic/phenolic interactions
2-Nitrophenol	88.5 ± 1.3	29.5 ± 0.5	Slightly lower	Intramolecular H-bonding reduces surface interaction
Phenol	72.4 ± 1.6	24.1 ± 0.6	Lower	Weaker polarity; no nitro group
1:1:1 NP isomer mixture	84.0 ± 1.5	84.0 ± 1.8 (total)	Competitive	Shared aromatic sites; 4-NP preferred
4-NP + phenol mixture	90.5 ± 1.2	60.3 ± 1.0	4-NP retained preferentially	Nitro group enhances affinity

### Preliminary economic analysis and sustainability metrics

A preliminary economic analysis estimated the production cost of the P(NaSS-co-AA)/AC composite to be approximately USD 5.22 kg⁻¹, based on the experimental inputs (Ficus leaves, H₃PO₄, NaSS, acrylic acid, KPS, ethanol, energy, and labor) (Table 10). This cost is competitive with commercial functionalized polymers (USD 20–50 kg⁻¹) and comparable to premium activated carbons (USD 5–15 kg⁻¹), while providing enhanced adsorption performance toward 4-nitrophenol. This estimate is based on laboratory-scale production and is expected to decrease further upon process optimization and scale-up.

The composite also demonstrated favorable sustainability metrics, including a 60 wt% biomass-derived content, 90.1% adsorption performance retention after five regeneration cycles, and 86.6% performance retention in real wastewater, supporting its potential for practical and sustainable water treatment.

*Ficus* leaves were selected because they are abundant waste biomass, possess high lignocellulosic content suitable for activated carbon production, provide an activated carbon yield of approximately 35%, and have been successfully used as precursors for environmental remediation.

**Table 10:** Preliminary production cost estimation for the P(NaSS-co-AA)/AC composite.

Input	Amount per kg composite	Unit cost (USD)	Cost (USD kg⁻¹)
Dried *Ficus* leaves	3.0 kg	0.05/kg	0.15
H₃PO₄ (80% recovery)	0.18 kg	2.00/kg	0.36
NaSS monomer	0.29 kg	8.00/kg	2.32
Acrylic acid	0.12 kg	1.60/kg	0.19
KPS initiator	0.006 kg	4.00/kg	0.02
Ethanol (recovered)	0.08 L	1.20/L	0.10
Process energy	12 kWh	0.10/kWh	1.20
Labor and overhead	–	–	0.87
Total estimated production cost	–	–	~ 5.22

Sustainability indicators: Biomass-derived content = 60 wt%; adsorption capacity retention after five regeneration cycles = 90.1%; performance retention in real wastewater = 86.6%.

### Preliminary fixed-bed column study and scale-up considerations

To evaluate the feasibility of continuous-flow operation, preliminary fixed-bed column breakthrough experiments were conducted (C₀ = 50 mg L⁻¹, bed mass = 2.0 g, flow rate = 2 mL min⁻¹, bed volume = 8 mL, pH = 7). Breakthrough (C/C₀ = 0.05) occurred after approximately 240 min (~ 60 bed volumes), while column exhaustion (C/C₀ = 0.95) was reached after approximately 600 min (~ 150 bed volumes). These results demonstrate the potential of the P(NaSS-co-AA)/AC composite for continuous wastewater treatment and provide a basis for future Thomas, Yoon–Nelson, and BDST model analyses for process scale-up.

Several engineering challenges should be considered for large-scale implementation. Achieving a uniform polymer coating on activated carbon at higher production volumes requires optimized mixing and viscosity control. Recovery and recycling of ethanol and H₃PO₄ (target recovery ≈ 80%) are essential to improve process economics and sustainability. Long-term operation will require efficient regeneration protocols, including desorbent management and treatment of spent regenerant solutions. In addition, appropriate strategies for thermal regeneration or environmentally safe disposal of the spent adsorbent, which contains approximately 60 wt% carbon, should be implemented. These challenges are common for polymer–carbon composite adsorbents and can be addressed through established packed-bed process design and engineering practices, supporting the potential for future industrial wastewater treatment applications.

### Comparison of 4-nitrophenol removal efficiency across different adsorbents

Table 11 summarizes the adsorption capacities of different adsorbents for 4-nitrophenol (4-NP) under their respective optimal conditions. Among the tested materials, the P(NaSS-co-AA)/AC composite derived from Ficus leaves exhibited an adsorption capacity of 65.0 mg/g at neutral pH (7.0). This adsorption performance could be attributed to the synergistic combination of high-surface-area activated carbon and functional groups from the P(NaSS-co-AA) polymer, which together facilitates multimodal interactions, including π–π stacking, hydrogen bonding, and electrostatic attraction.

In comparison, conventional polymers such as P(AN-co-AA) with thioamide achieve slightly lower adsorption (58.4 mg/g) under mildly acidic conditions (pH 6.0), indicating that the P(NaSS-co-AA)/AC composite can operate effectively under neutral conditions. Natural minerals such as bentonite and graphene-based composites such as GO-ATT also display moderate adsorption capacities (40–46 mg/g), while reduced graphene oxide (rGO) and multi-walled carbon nanotubes (MWCNTs) show lower capacities (4.4–15.5 mg/g) under their respective reported experimental conditions. Regarding adsorption kinetics, the equilibrium contact time of the P(NaSS-co-AA)/AC composite was 120 min, which is comparable to those reported for GO-ATT, MIL-68(Al)/reduced graphene oxide, and DMAEMA-grafted cellulose (CD-MIP), and notably shorter than that of natural bentonite (240 min) and the collagen-based magnetic double-network hydrogel (48 h). Although some adsorbents reached equilibrium within 60 min, they exhibited lower or comparable adsorption capacities than the present composite.

Overall, the adsorption capacity obtained in this study demonstrates competitive performance compared with several reported adsorbents for 4-NP removal. While the observed capacity is comparable to those of other polymeric adsorbents reported in the literature, the present composite offers effective adsorption under neutral pH conditions and benefits from the synergistic combination of activated carbon and functionalized polymeric components. Recent studies have also highlighted the growing interest in advanced polymeric and hydrogel-based adsorbents for water treatment applications due to their tunable surface chemistry and abundant adsorption sites^59–62^.

**Table 11:** Comparative adsorption capacities of various adsorbents for 4-Nitrophenol.

Material Composition	q_max_ (mg/g)	Optimal pH	Equilibrium Time	Kinetics	Reference
P(NaSS-co-AA)/AC (Ficus Leaves)	65.0	7.0	120 min	PSO	This Study
P(AN-co-AA) with thioamide	58.4	6.0	60 min	PSO	^64^
Natural Bentonite	46.4	7.0	240 min	PSO	^65^
GO-ATT (Graphene Oxide)	40.0	7.0	120 min	PSO	^66^
MIL-68(Al)/Reduced Graphene Oxide composite	15.5	6.0	120 min	PSO	^67^
Multi-walled CNTs	4.4	6.0	60 min	PSO	^68^
Collagen-(AMPS-MAA/AAm)-Fe_3_O_4_@SiO_2_ magnetic double-network nanocomposite hydrogel (DNNH)	19.20	7.0	48 h	PSO	^69^
DMAEMA-grafted cellulose (CD-MIP)	26.87	7.0	120 min	PSO	^70^

## Conclusion

This research reports the synthesis of a novel Ficus-leaf-derived P(NaSS-co-AA)/AC composite for 4-nitrophenol removal, achieving a maximum adsorption capacity of 65.0 ± 1.25 mg g⁻¹. The composite demonstrated competitive adsorption performance, supported by the combined contribution of activated-carbon domains and functional polymeric groups. Adsorption is best described by the Langmuir isotherm (R² = 0.994, RMSE = 0.85 ± 0.08) and pseudo-second-order kinetics (R² = 0.999, RMSE = 0.47 ± 0.04), indicating monolayer chemisorption. The adsorption mechanism was supported by FTIR and XPS evidence rather than inferred solely from pseudo-second-order kinetics. FTIR showed new –NO₂ bands and shifts in –SO₃⁻ and –COO⁻ groups, while XPS confirmed 4-NP uptake through the N 1s signal at ~ 406 eV. These results support combined π–π interactions, hydrogen bonding, electrostatic attraction, pore filling, and possible surface complexation. Optimal conditions were pH 7.0, 1.5 g/L adsorbent and 120-minute contact time. The material’s endothermic adsorption, mesoporous structure, and large surface area enhance performance. Utilizing Ficus leaves as a low-cost, eco-friendly carbon source offers environmental and economic advantages. The specific novelty of this work lies in the combination of Ficus leaf-derived AC with the bifunctional P(NaSS-co-AA) copolymer bearing simultaneously –SO₃⁻ and –COO⁻ groups, yielding superior performance (65.0 ± 1.25 mg/g) over either component alone and over comparable polymer–carbon composites in the literature. Limitations of the current study and future research directions: (1) All adsorption experiments were conducted in batch mode; column/continuous-flow operation requires further optimization. (2) Regeneration was tested for only 5 cycles; long-term stability (> 20 cycles) requires investigation. (3) Real wastewater testing was conducted at laboratory scale only; pilot-scale trials are needed to validate performance under industrial conditions. (4) The quantitative contributions of individual interaction modes (electrostatic vs. H-bonding vs. π–π) to the overall adsorption capacity have not been deconvoluted; DFT molecular modeling or selective blocking experiments are recommended for future work. (5) Economic analysis is preliminary and based on laboratory-scale inputs; industrial-scale cost estimation requires pilot-plant data. Future research should address pilot-scale testing, extended regeneration (> 20 cycles), real industrial wastewater trials under continuous-flow conditions, DFT modeling of molecular interactions, and life-cycle assessment to fully evaluate the sustainability potential of this composite.

## Data Availability

Data Will be available upon request.
